# HASEL Artificial Muscles for a New Generation of Lifelike Robots—Recent Progress and Future Opportunities

**DOI:** 10.1002/adma.202003375

**Published:** 2020-11-09

**Authors:** Philipp Rothemund, Nicholas Kellaris, Shane K. Mitchell, Eric Acome, Christoph Keplinger

**Affiliations:** ^1^ Department of Mechanical Engineering University of Colorado, Boulder 1111 Engineering Drive Boulder CO 80309 USA; ^2^ Materials Science and Engineering Program University of Colorado, Boulder, Sustainability Energy & Environment Community Boulder CO 80303 USA

**Keywords:** artificial muscles, electrostatics, HASEL actuators, robotics, soft actuators

## Abstract

Future robots and intelligent systems will autonomously navigate in unstructured environments and closely collaborate with humans; integrated with our bodies and minds, they will allow us to surpass our physical limitations. Traditional robots are mostly built from rigid, metallic components and electromagnetic motors, which make them heavy, expensive, unsafe near people, and ill‐suited for unpredictable environments. By contrast, biological organisms make extensive use of soft materials and radically outperform robots in terms of dexterity, agility, and adaptability. Particularly, natural muscle—a masterpiece of evolution—has long inspired researchers to create “artificial muscles” in an attempt to replicate its versatility, seamless integration with sensing, and ability to self‐heal. To date, natural muscle remains unmatched in all‐round performance, but rapid advancements in soft robotics have brought viable alternatives closer than ever. Herein, the recent development of hydraulically amplified self‐healing electrostatic (HASEL) actuators, a new class of high‐performance, self‐sensing artificial muscles that couple electrostatic and hydraulic forces to achieve diverse modes of actuation, is discussed; current designs match or exceed natural muscle in many metrics. Research on materials, designs, fabrication, modeling, and control systems for HASEL actuators is detailed. In each area, research opportunities are identified, which together lays out a roadmap for actuators with drastically improved performance. With their unique versatility and wide potential for further improvement, HASEL actuators are poised to play an important role in a paradigm shift that fundamentally challenges the current limitations of robotic hardware toward future intelligent systems that replicate the vast capabilities of biological organisms.

## Graphical abstract

Hydraulically amplified self‐healing electrostatic (HASEL) actuators are a new, versatile class of high‐performance, self‐sensing artificial muscles. Current research in materials, designs, fabrication, modeling, and controls is discussed and the wide opportunities for future interdisciplinary research to realize HASEL artificial muscles with drastically improved performance for use as building blocks of bioinspired intelligent systems of the future are laid out.

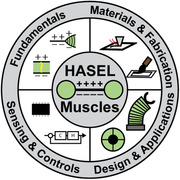

## 1 Introduction

Popular culture is rife with examples of robotic assistants that increase our productivity and enhance our quality of life—from aiding humans in dangerous areas such as space exploration, to helping with mundane tasks around the home. This vision of robots that are integral to our daily lives is multifaceted. In part, it is driven by immediate needs, such as addressing the increasing labor shortages in industries such as agriculture, where there are not enough people to harvest crops in time; in other areas, it is driven by the desire to surpass our physical limitations through the integration of humans and machines: wearable robotics or exoskeletons that surpass our innate strength and endurance, or provide assistance to the elderly or those rehabilitating from injury. While robots today are impressive in their capabilities—especially in controlled environments such as an assembly line in a factory—they are often ill‐suited in unstructured environments and can be dangerous for humans in collaborative situations. In order to realize a future in which robots can adapt to changing tasks and environments, we need machines that are extremely versatile in terms of their decision making and that are based on robotic hardware that can dynamically respond in a variety of situations.

In the last decades advances in robotics have been driven predominantly by increases in computing power, new sensing concepts and control algorithms, new machine learning approaches, and more generally artificial intelligence. Less focus has been placed on improving the body of robots, so they continue to rely on rigid, mostly metallic components and electromagnetic actuators such as servo and stepper motors. Ample inspiration for the search of new materials, actuation mechanisms, and sensors in robots can be found in nature. Using a variety of soft materials such as muscle and skin, nature has produced organisms that drastically outperform current robots in dexterity, agility, and adaptability. Especially inspirational is the natural muscle, which has evolved into an extremely versatile actuator. Natural muscle enables the rapid wing‐flapping rates of a hummingbird, it is strong enough to move an elephant, and it enables the complex motion of the arm of an octopus, whose versatility is still unmatched by human‐made machines. Additionally, muscle continuously regenerates over the lifetime of an organism, heals after damage, and is seamlessly integrated with nerves for sensing.^[^ ^1^ ^]^

**Figure 1:**
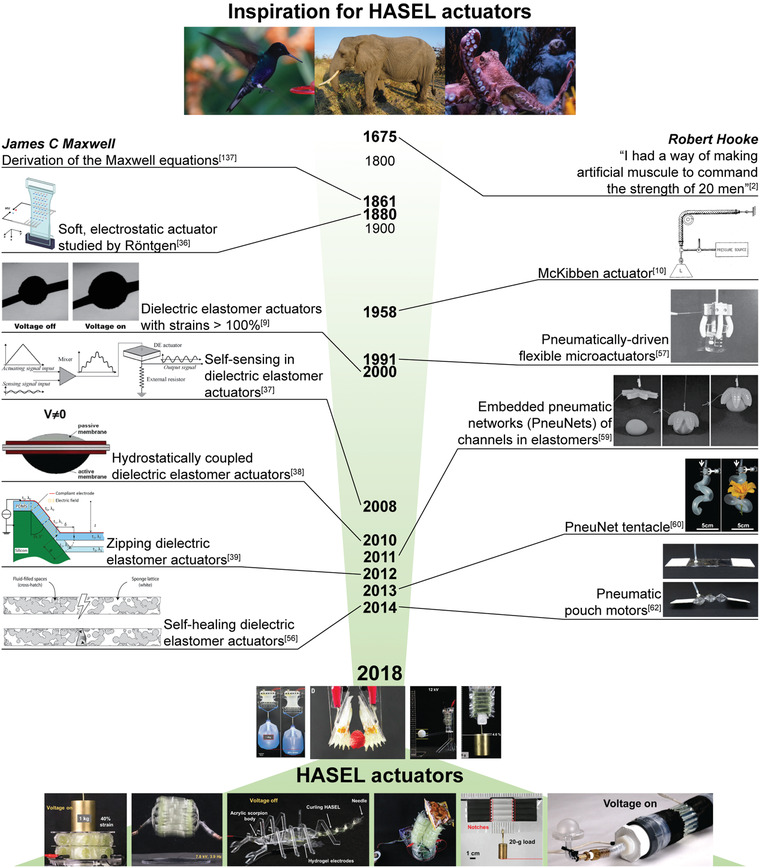
Selected sources of inspiration for hydraulically amplified self‐healing electrostatic (HASEL) actuators. The timeline shows key sources of inspirations for the invention of the HASEL technology. Photo of the hummingbird and elephant by Günter Oesterling and reproduced with permission. Photo of the octopus by Jeahn Laffitte on Unsplash and reproduced with permission. “Soft, electrostatic actuator studied by Röntgen” image: Reproduced with permission.^[^ ^137^ ^]^ Copyright 2010, National Academy of Sciences USA. “McKibben actuator” image: Reproduced from ref. ^[^ ^10^ ^]^. “Pneumatically driven flexible microactuators” image: Reproduced with permission.^[^ ^59^ ^]^ Copyright 1996, Elsevier Ltd. “Dielectric elastomer actuators with strains > 100%” image: Reproduced with permission.^[^ ^9^ ^]^ Copyright 2000, AAAS. “Self‐sensing in dielectric elastomer actuators” image: Reproduced with permission.^[^ ^38^ ^]^ Copyright 2008, Elsevier Ltd. “Hydrostatically coupled dielectric elastomer actuators” image: Reproduced with permission.^[^ ^39^ ^]^ Copyright 2010, IEEE. “Embedded pneumatic networks (PneuNets) of channels in elastomers” image: Reproduced with permission.^[^ ^60^ ^]^ Copyright 2011, Wiley‐VCH. “Zipping dielectric elastomer actuators” image: Reproduced with permission.^[^ ^40^ ^]^ Copyright 2012, SPIE. “PneuNet tentacle” image: Reproduced with permission.^[^ ^61^ ^]^ Copyright 2013, Wiley‐VCH. “Self‐healing dielectric elastomer actuators” image: Reproduced with permission.^[^ ^57^ ^]^ Copyright 2014, American Institute of Physics. “Pneumatic pouch motors” image: Reproduced with permission.^[^ ^63^ ^]^ Copyright 2014, IEEE. “HASEL actuators” top row images; two left images: Reproduced with permission.^[^ ^14^ ^]^ Copyright 2018, The Authors, published by AAAS; Two right images: Reproduced with permission.^[^ ^15^ ^]^ Copyright 2018, The Authors, published by AAAS. “HASEL actuators” bottom row, left four images: Reproduced under the terms of the CC‐BY Creative Commons Attribution 4.0 International license (https://creativecommons.licenses/by/4.0).^[^ ^89^ ^]^ Copyright 2019, The Authors, published by Wiley‐VCH. “HASEL actuators” bottom row, right two images: Reproduced with permission.^[^ ^97^ ^]^ Copyright 2019, Wiley‐VCH.

Actuators are a key component of all robotic systems. Not surprisingly, the remarkable capabilities of muscle have inspired scientists and engineers for centuries (**Figure** **1**). Already in the 17th century, Robert Hooke experimented with gunpowder in the attempt to replicate the performance of natural muscle.^[^ ^2^ ^]^ Evidently, Robert Hooke's efforts were unsuccessful, and creating actuators that truly match all performance metrics of mammalian skeletal muscle simultaneously—such as actuation strains of 40%,^[^ ^3^ ^]^ strain rates of 500% s^−1^,^[^ ^3^ ^]^ actuation stresses of 0.35 MPa,^[^ ^4^ ^]^ energy densities of 40 J kg^−1^,^[^ ^4^ ^]^ and efficiencies of 40%^[^ ^3^ ^]^—remains a grand challenge to this day.^[^ ^5^ ^]^ For example, the July 2019 issue of *Science* featured three separate research articles dedicated to the development of artificial muscles.^[^ ^6^, ^7^, ^8^ ^]^ Today, a broad range of artificial muscle actuators exists, including dielectric elastomer actuators (DEAs),^[^ ^9^ ^]^ fluid‐driven soft actuators,^[^ ^10^ ^]^ ionic polymer‐metal composites,^[^ ^11^ ^]^ shape memory alloys,^[^ ^12^ ^]^ and thermally responsive fiber‐based muscles.^[^ ^13^ ^]^ Many of these types of actuators excel in specific performance metrics, but exhibit poor performance in others.

Herein, we discuss the background, fundamentals, recent progress (including articles appearing online before April 7, 2020), and future research opportunities for hydraulically amplified self‐healing electrostatic (HASEL) artificial muscles.^[^ ^14^, ^15^ ^]^ HASEL artificial muscles are a versatile new technology that couples electrostatic and hydraulic forces to achieve excellent overall muscle‐like performance, the ability to self‐sense their state of deformation, and the ability to self‐heal from electrical damage. HASEL actuators were first introduced in 2018 by our research group,^[^ ^14^, ^15^ ^]^ and in this progress report we provide our point of view on the development of HASEL actuators, with the overall intention to provide a comprehensive discussion that will stimulate future research on the HASEL technology but also more generally to help accelerate the development of “robotic materials”—a new class of materials systems that tightly integrate actuation, sensing and even computation to provide physical building blocks for the intelligent systems of the future.

While we include background on artificial muscle technologies that inspired HASEL actuators, this is not a comprehensive review of the fields of artificial muscles or soft robotics. Interested readers are referred to other articles, which discuss additional specific aspects or give a more comprehensive overview of these fields of research.^[^ ^3^, ^4^, ^16^, ^17^, ^18^, ^19^, ^20^, ^21^, ^22^, ^23^, ^24^, ^25^, ^26^, ^27^, ^28^, ^29^, ^30^, ^31^, ^32^, ^33^, ^34^, ^35^ ^]^

### 1.1 Inspiration for HASEL Artificial Muscles

Since Robert Hooke's early attempts, many different types of artificial muscles have been investigated. However, researchers have been unsuccessful in creating an artificial muscle that matches the performance of natural muscle in all metrics. The invention of HASEL actuators began with a desire to create a new type of artificial muscle that combines the benefits of two well‐known technologies—dielectric elastomer actuators (DEAs) and fluid‐driven soft actuators—while also addressing their shortcomings.

#### 1.1.1 Dielectric Elastomer Actuators

In the middle of the 19th century James C. Maxwell derived the so‐called Maxwell equations,^[^ ^36^ ^]^ which laid the foundations for classical electromagnetism. The Maxwell equations showed that electric fields induce mechanical stresses in matter (the Maxwell stress). In 1880, Röntgen sprayed electric charges onto a strip of natural rubber; he observed that the electric field between the charges induced a Maxwell stress in the rubber which caused the rubber to increase in length, thereby discovering the underlying principle of dielectric elastomer actuators (DEAs).^[^ ^37^ ^]^ However, it was not until 2000 that DEAs gained widespread interest in the research community, when Pelrine et al.^[^ ^9^ ^]^ discovered that prestretching DEA materials enables voltage‐controlled actuation strains >100%. The seminal work by Pelrine et al. motivated many researchers to understand and improve the performance of DEAs, as large actuation strains combined with the benefits of electrical control make DEAs a compelling technology for soft actuators.

DEAs allow for close integration of actuation and sensing—analogous to the proprioceptive capabilities of natural muscle.^[^ ^1^ ^]^ Since DEAs are deformable capacitors, the state of deformation of a DEA can be determined by measuring its capacitance. Jung et al.^[^ ^38^ ^]^ showed that both sensing and actuation signals for DEAs can be combined into a single input to enable self‐sensing of actuation. While many configurations of DEAs have been explored, two types in particular have influenced the invention of HASEL actuators: hydrostatically coupled DEAs^[^ ^39^ ^]^ in which liquids couple the deformation of multiple elastomer membranes and DEA‐based pumps in which electrostatic “zipping” of dielectric elastomer membranes onto rigid substrates is used to pump fluids.^[^ ^40^, ^41^ ^]^

Despite the convenience of electrical control and excellent electromechanical performance, DEAs usually need a prestretch^[^ ^9^, ^42^ ^]^ and rigid frames to achieve large strains, or stacked configurations to achieve linear, muscle‐like contraction upon activation;^[^ ^43^, ^44^ ^]^ these constraints limit the design freedom of DEAs. Further, DEAs require stretchable material systems for dielectric layers and electrodes.^[^ ^45^, ^46^, ^47^ ^]^ Elastomer membranes and stretchable electrodes often suffer from reduction in electrical performance, mechanical fatigue, and degradation under high strains.^[^ ^45^, ^48^, ^49^, ^50^, ^51^ ^]^ Reliability is a critical challenge to up‐scaling of DEAs for practical applications, such as humanoid robots, as the elastomer layers are prone to catastrophic failure by dielectric breakdown at material defects, and the fabrication of large, thin, defect‐free elastomer membranes is challenging.^[^ ^48^, ^52^, ^53^, ^54^ ^]^ One approach to address this problem is the use of self‐clearing electrodes, which electrically insulate the damaged zones of the elastomer.^[^ ^53^, ^55^, ^56^ ^]^ Industrial high voltage transformers use liquid dielectrics that immediately return to an insulating state after dielectric breakdown. Hunt et al.^[^ ^57^ ^]^ used this principle to build a self‐healing DEA based on a foam swollen with a liquid dielectric. When damaged (due to breakdown or puncturing) the liquid dielectric flowed into the damaged zone and resealed the dielectric layer. The work by Hunt et al. and the self‐healing capabilities of industrial high voltage transformers served as an inspiration for the self‐healing mechanism in HASEL actuators.

#### 1.1.2 Fluid‐Driven Soft Actuators

The first type of soft pneumatic actuator was introduced in 1958 by Gaylord.^[^ ^10^ ^]^ These so‐called McKibben actuators consist of a soft tube wrapped in a fiber mesh and linearly contract when pressurized with a fluid. In the 1990s, Suzumori et al.^[^ ^58^, ^59^ ^]^ demonstrated more complex modes of actuation with pneumatically‐driven microactuators, in which multiple pneumatic chambers were combined into a single fiber reinforced tube. Wide development of soft pneumatic actuators began in earnest in 2010; with the advent of rapid manufacturing tools such as 3D printing, actuators based on pneumatic networks (PneuNets) in elastomers could be easily fabricated.^[^ ^60^ ^]^ PneuNets can be designed for a variety of modes of actuation and have led to a diverse range of soft robot configurations and capabilities.^[^ ^60^, ^61^, ^62^ ^]^ In contrast to the elastomeric materials used for PneuNets, pouch motors or “Peano” actuators^[^ ^63^ ^]^ consist of pouches made from thin, inextensible plastic films. When inflated with pressurized air, the pouch changes shape, which (depending on orientation and constraints) can be used for contraction, expansion, or bending.

Whereas fluid‐driven soft actuators offer a large design freedom and versatile modes of actuation, the need for a source of pressurized fluid (e.g., compressors, pumps, or pressurized reservoirs of fluid) is a distinct disadvantage. Additionally, valves are required to regulate flow into and out of the actuators. Therefore, fluid‐driven soft actuators are often tethered.^[^ ^64^, ^65^, ^66^ ^]^ Untethered, mobile, fluid‐driven soft robots have been realized,^[^ ^67^ ^]^ but the capabilities of mobile pumps are limited, and the need for valves leads to bulky and heavy control systems for robots with multiple actuators. Flow control has been successfully integrated directly into the structure soft actuators,^[^ ^68^, ^69^, ^70^, ^71^ ^]^ but attempts to integrate a pressurized fluid‐reservoir into the soft structure have led to limited performance.^[^ ^71^ ^]^ Finally, the losses associated with flow of fluids through tubing and valves limit the speed and reduce the efficiency of fluid‐driven soft actuators.

### 1.2 Fundamental Principles of HASEL Artificial Muscles

**Figure 2:**
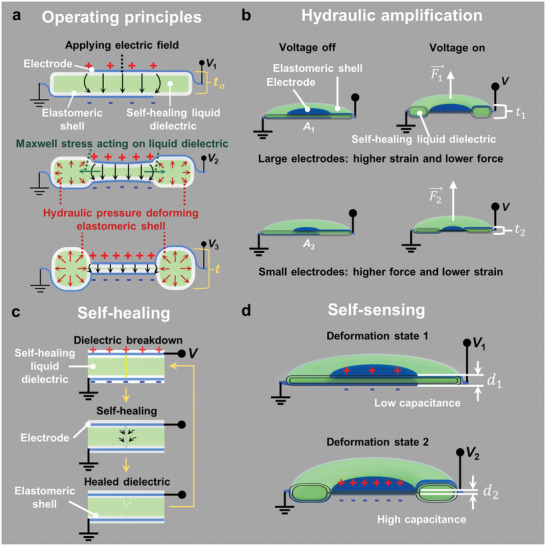
Basic operating principles of HASEL actuators. a) HASEL actuators consist of polymer shells that are coated with opposing electrodes and that are filled with a liquid dielectric. When a voltage is applied to the actuator an electric field arises between the electrodes. The electric field causes a Maxwell stress in the actuator, which leads to redistribution of the liquid dielectric and deformation of the actuator. b) HASEL actuators use the principles of hydraulic amplification to generate forces. c) After a dielectric breakdown event, the liquid dielectric redistributes and returns to its insulating state. This property leads to electrical self‐healing in HASEL actuators. d) The electrodes of HASEL actuators form a capacitor. Shape changes due to applied voltages or external forces can be detected by measuring the capacitance. a–d) Adapted with permission.^[^ ^14^ ^]^ Copyright 2018, The Authors, published by AAAS.

A basic HASEL actuator consists of a deformable shell (flexible or stretchable) that is covered with a pair of opposing electrodes and filled with a liquid dielectric (**Figure** **2**a).^[^ ^14^ ^]^ When voltage is applied to the electrodes, a Maxwell stress acts on the shell and the liquid dielectric, thereby driving local redistribution of the liquid dielectric, which results in a shape change of the soft hydraulic structure. This working principle enables HASEL actuators to use the principles of hydraulic amplification (Pascal's law) (Figure 2b). Changing the size of the electrode with respect to the size of the shell modifies stroke and force output (analogous to a hydraulic system of pistons); a large electrode results in larger actuation strain but lower force output whereas an actuator with small electrodes results in a smaller actuation strain but larger force output. The use of liquid dielectrics also enables self‐healing from dielectric breakdown to improve reliability (Figure 2c); when dielectric breakdown occurs, the liquid redistributes and returns to an insulating state. Additionally, HASEL actuators possess the ability to self‐sense their state of deformation (Figure 2d). The structure of a HASEL actuator is that of a deformable capacitor. When the actuator deforms due to an external force or applied voltage, the size and spacing of the electrodes changes, resulting in a measurable change of capacitance that is a function of the state of deformation of the actuator.

Overall, the fundamental principles of HASEL actuators are inspired by the versatility of fluid‐driven soft actuators as well as the performance and electrical control of DEAs. HASEL actuators use electrostatic forces to locally generate hydraulic pressure in soft structures that are filled with liquid dielectrics. This approach combines the benefits of both DEAs and fluid‐driven soft actuators, while also addressing their challenges. The use of electrostatic forces allows for fast and efficient activation. Because a fluid is used to induce deformation, HASEL actuators can be designed for a variety of actuation modes, whereas the local generation of pressure avoids the need for valves and tubing. The use of liquid dielectrics also improves reliability and allows up‐scaling, as liquid dielectrics can self‐heal from dielectric breakdown. Additionally, many designs of HASEL actuators do not require dielectrics and electrodes that are stretchable, but they can be built from materials that are only flexible instead of stretchable; flexible materials are a much larger class of materials than stretchable materials and include high‐performance electrical insulators (e.g., poly(vinylidene fluoride) terpolymer)^[^ ^72^ ^]^ and low‐resistance conductors (e.g., thin metal films or conductive silver inks).

### 1.3 Outline of the Progress Report

The Progress Report is organized in topical sections and loosely guided by the order in which the technology developed. Most sections include a summary that describes specific problems and possible avenues for future research. Sections 2 and 3 describe different designs of HASEL actuators whose shells are made with elastomeric or thermoplastic materials, respectively. Section 3 also presents a method for rapid prototyping of thermoplastic actuators. Section 4 discusses the demonstrated performance metrics of HASEL actuators. Section 5 describes the first modeling efforts to understand the electromechanical behavior of HASEL actuators. Section 6 discusses several pathways for substantially improving the performance of HASEL actuators. Section 7 discusses closely related technologies. Section 8 describes high voltage electronics, sensing, and control of HASEL actuators toward implementation in untethered soft robotic systems. Section 9 discusses the significance of HASEL actuators for a new generation of lifelike robots.

## 2 HASEL Artificial Muscles Made from Elastomers

The first HASEL actuators utilized silicone elastomers, a common material in the field of soft robotics, because they are highly stretchable, resilient to damage, and commercially available. Additionally, silicone elastomers have good dielectric properties (relative permittivity ≈3 and dielectric breakdown strength ≈50–120 kV mm^−1^)^[^ ^73^, ^74^ ^]^ which are important for soft electrostatic actuators. There is also an active community of researchers who are developing elastomers that are specialized for electromechanical applications, and that have unique properties such as self‐healing from mechanical damage.^[^ ^75^, ^76^, ^77^, ^78^ ^]^ The next sections discuss two distinctly different designs, which were created using elastomeric materials: elastomeric donut HASEL actuators (Section 2, .1) and planar HASEL actuators (Section 2, .2). Section 2, .3 describes opportunities for research on HASEL actuators made from elastomeric materials.

### 2.1 Elastomeric Donut HASEL Actuator

**Figure 3:**
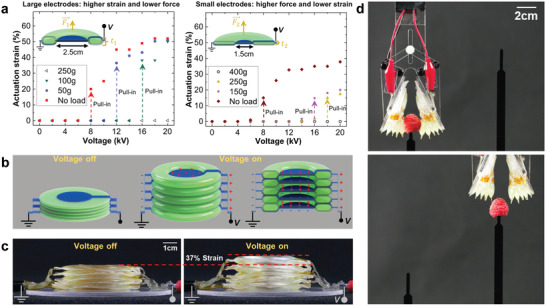
The elastomeric donut HASEL actuator. a) The elastomeric donut HASEL actuator consists of a circular elastomeric pouch that is filled with a liquid dielectric and partially covered on both sides with circular electrodes. When a voltage is applied to the actuator, it deforms into a donut shape (see also Figure 2). The strain–voltage curve of a donut HASEL actuator shows a pronounced pull‐in instability which leads to distinct off‐ and on‐states. The output force and actuation strain of an elastomeric donut HASEL actuator follow the principles of hydraulic amplification: actuators with large electrodes exhibit larger actuation strains but generate lower forces than actuators with small electrodes. b) Multiple actuators can be stacked to increase the total actuation stroke. c) Photographs of a stack of five elastomeric donut HASEL actuators in the off‐ and on‐states. d) Photographs of a soft gripper, whose design was based on elastomeric donut HASEL actuators. The gripper is gentle enough to handle raspberries without damaging them. a–d) Reproduced with permission.^[^ ^14^ ^]^ Copyright 2018, The Authors, published by AAAS.

The elastomeric donut HASEL actuator^[^ ^14^ ^]^ is comprised of a circular shell filled with a liquid dielectric (Figure 2a,b). A pair of circular electrodes with a smaller diameter than the shell is placed concentrically on both sides of the shell. When voltage is applied to the electrodes, electrostatic forces attract the electrodes to each other and displace liquid to the edge of the shell which deforms the actuator into a “donut” shape and results in an increase in thickness (Figure 2a,b). Elastomeric donut HASEL actuators exhibit a pronounced pull‐in instability (**Figure** **3**a).^[^ ^14^ ^]^ At low voltages deformation is small, because the electrostatic forces cannot overcome the hydrostatic pressure in the liquid dielectric and the stiffness of the shell. At a critical voltage, the electrostatic forces overcome the resistance in the actuator and a positive feedback between the deformation and the electrostatic forces (a decrease of the distance between the electrodes increases the electrostatic forces)^[^ ^79^, ^80^ ^]^ leads to a sudden pull‐in instability (Figure 3a). Actuation voltages beyond the pull‐in instability again lead to a gradual deformation. Donut HASEL actuators achieved large actuation strains (>50% at a load of 0.5 N and a voltage of 20 kV). Following the principles of hydraulic amplification, changing the size of the electrodes leads to different force and displacement characteristics (Figure 3a). The liquid dielectric inside the shell also allowed the actuators to electrically self‐heal repeatedly from dielectric breakdown.^[^ ^14^ ^]^ The large change in capacitance during deformation may be used to self‐sense the deformation of the elastomeric donut HASEL actuator (Section 8, .3). Thus, donut HASEL actuators exhibit all basic properties of HASEL actuators shown in Figure 2.

Elastomeric donut HASEL actuators do not require rigid frames, which simplifies combining multiple actuators for increased stroke and force and configuring them for different types of actuation. By stacking multiple actuators in series, the actuation stroke can be increased (Figure 3b,c). Placing several elastomeric donut HASEL actuators in parallel increases the force output. Finally, stacks of elastomeric donut HASEL actuators can also be configured to achieve new types of actuation. For example, we constrained a stack of actuators on one side to create a soft gripper capable of handling delicate objects (Figure 3d).^[^ ^14^ ^]^

### 2.2 Planar HASEL Actuator

**Figure 4:**
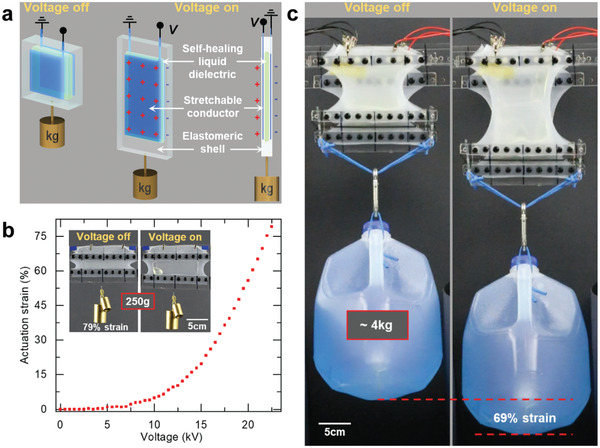
The planar HASEL actuator. a) The planar HASEL actuator consists of a planar elastomeric shell which encloses a chamber that is filled with a liquid dielectric. Stretchable electrodes cover both sides of the elastomeric shell over the entire area of the chamber. The actuator is prestretched perpendicular to the direction of actuation. When a voltage is applied to the electrodes, the thickness of the actuator decreases, and the actuator elongates. b) Demonstration of quasi‐static linear actuation with a planar HASEL actuator. c) Six planar HASEL actuators dynamically lifting a gallon of water. a–c) Reproduced with permission.^[^ ^14^ ^]^ Copyright 2018, The Authors, published by AAAS.

A second type of elastomeric HASEL actuators form a planar geometry wherein electrodes cover most of the region containing the liquid dielectric (**Figure** **4**a).^[^ ^14^ ^]^ Applying a voltage to the electrodes causes a decrease in the thickness and increase in area (Figure 4a), similar to DEAs. However, because part of the dielectric layer is liquid instead of solid, the stiffness of planar HASEL actuators is smaller than DEAs of the same dimensions and the same solid dielectric material. Consequently, planar HASEL actuators can achieve higher actuation strains than DEAs at the same voltage.^[^ ^14^ ^]^ In a specific design for a linearly expanding planar HASEL actuator (Figure 4a,b), a rectangular actuator is prestretched perpendicular to the actuation direction and fixed to rigid frames, a geometry inspired by laterally constrained DEAs.^[^ ^81^ ^]^ At a tensile load of 2.5 N, this actuator elongated by 79% when a voltage of 22.5 kV was applied (Figure 4b). When operated near resonance, these actuators exhibit large actuation strains (>100%) and high specific power outputs (614 W kg^−1^ peak).^[^ ^14^ ^]^ Six planar HASEL actuator could lift a gallon of water (Figure 4c). Additionally, the structure of planar HASEL actuators allows capacitive sensing of their deformation, which is discussed in Section 8, .3.

### 2.3 Research Opportunities for HASEL Actuators Made from Elastomers

The initial experiments with elastomeric HASEL actuators revealed several interesting characteristics that could be explored in future research. The pull‐in instabilities of donut HASEL actuators may be exploited for applications that require bistable operation or large and rapid response to small changes in input. Inspired by unique actuation schemes in nature, bistable actuation has been utilized successfully in other soft actuator systems.^[^ ^69^, ^82^, ^83^ ^]^ The electrode placement and geometry in a donut HASEL actuator can be modified to achieve new types of functionalities, such as tunable lenses.^[^ ^84^ ^]^ The large dynamic linear actuation strains and high power‐to‐weight ratios of planar HASEL actuators are compelling for applications requiring periodic motion. Additionally, the ability to store elastic energy within the elastomeric shell of planar HASEL actuators may enable efficient and high‐power dynamic motions, which could be applied to legged locomotion or flight. Developing theories for both static and dynamic characteristics of elastomeric HASEL actuators could aid in the design of actuators for specific applications (Section 5).

There are many materials‐related opportunities for further development of elastomeric HASEL actuators. So far, thick layers (0.1–1 mm thick) of commercially available silicone elastomers have been used as the solid dielectrics of elastomeric HASEL actuators, as they are robust and easy to handle. Fabrication techniques for reliably producing HASEL actuators with thin elastomer layers (0.01–0.1 mm) would allow for operation at lower voltages. Further, efforts to produce high‐quality elastomer films in a particulate‐free environment would benefit the lifetime of elastomeric HASEL actuators, where dielectric breakdown is often driven by defects in the film layer. Self‐healing from dielectric breakdown was successfully demonstrated, but there are opportunities for improvement. Even though HASEL actuators functionally recover from dielectric breakdown, each breakdown event leads to the formation of gas in the liquid dielectric, and the elastomeric shells remain damaged (holes in the shells do not self‐heal, but self‐seal thus preventing liquid‐dielectric from leaking out). Repeated breakdown events may therefore result in regions of lower dielectric strength in the actuator and thus reduce performance. Incorporating elastomers which mechanically self‐heal from damage as well as gas‐permeable elastomers which allow for gas bubbles to quickly clear from the liquid dielectric region would improve the self‐healing performance of HASEL actuators.

## 3 HASEL Artificial Muscles Made from Thermoplastic Polymers

By coupling electrostatic and hydraulic actuation, the elastomeric HASEL actuators described above addressed many of the shortcomings of DEAs. However, the use of elastomer‐based materials systems left several limitations unsolved, including limited material choice—with the need for high‐quality elastomeric films and stretchable electrodes—and laborious fabrication procedures. A watershed moment for HASEL actuators came from the realization that most thermoplastic films are optimized for many of the properties needed for robust and capable HASEL actuators: they are easily processed, have excellent mechanical properties (e.g., packaging films), have outstanding electrical properties—thanks to the thin‐film capacitor industry—and are widely available. Many fluid‐driven soft actuators have used thermoplastic films for their mechanical properties and numerous available processing methods.^[^ ^66^, ^85^, ^86^, ^87^, ^88^ ^]^ Similarly, thin‐film dielectrics have been employed previously in electrostatic zipping actuators for their electrical properties.^[^ ^41^ ^]^

The introduction of thermoplastic materials systems avoided many of the drawbacks of stretchable dielectrics and electrodes such as limited material selection and device reliability.^[^ ^45^, ^48^, ^51^ ^]^ It also enabled a host of new actuation geometries, fabrication methods, and performance capabilities in HASEL actuators.^[^ ^15^, ^89^ ^]^ In the following sections, we outline progress in this area as it occurred, the implications of the results, and suggestions for future research.

### 3.1 Peano‐HASEL Actuator

**Figure 5:**
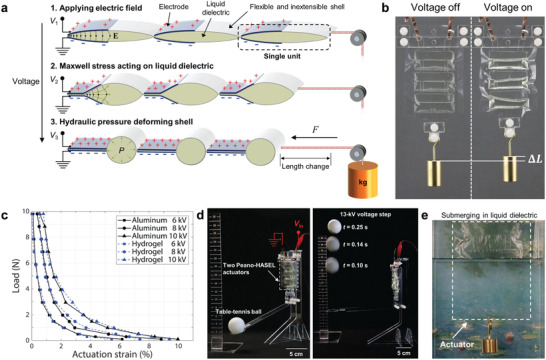
The Peano‐HASEL actuator. a) The Peano‐HASEL actuator is comprised of rectangular, inextensible but flexible shells. The shells are filled with a liquid dielectric and partially coated on both sides with flexible electrodes. When a voltage is applied to the actuator, the electrodes zip together from the edges of the shell where the electric field is highest, displacing the liquid dielectric. The liquid‐filled region of the shell takes a more cylindrical shape and the actuator contracts in length. b) Photographs of a Peano‐HASEL actuator lifting a weight. c) Prototypical Peano‐HASEL actuators demonstrated up to 10% linear contraction on activation, with both hydrogel and aluminum electrodes. d) Peano‐HASEL actuators allow for high‐speed actuation; two actuators connected to a lever arm throw a table tennis ball up into the air. e) When the actuator is made with transparent materials and submerged in an index matched fluid it becomes invisible. a–e) Reproduced with permission.^[^ ^15^ ^]^ Copyright 2018, The Authors, published by AAAS.

We first explored the concept of thermoplastic materials for HASEL actuators with the introduction of the Peano‐HASEL actuator, a soft electrostatic actuator capable of linear contraction on activation without relying on prestretch, rigid frames, or stacked configurations.^[^ ^15^ ^]^ Peano‐HASEL actuators combine the strengths of linearly contracting Peano‐fluidic actuators created by Niiyama et al.^[^ ^63^ ^]^ and Sanaan et al.,^[^ ^90^ ^]^ and the electrostatic principles of elastomeric HASEL actuators.^[^ ^14^ ^]^ The basic design of Peano‐HASEL actuators consists of rectangular shells, formed by bonding inextensible but flexible polymer films (**Figure** **5**a). These shells are filled with a liquid dielectric and sealed off to make a pouch; a pair of opposing electrodes is placed on either side of the pouch, covering a portion of the shell. On application of voltage between the electrodes, the resulting electric field causes the electrodes to zip together progressively, beginning from the edge of the shell where the electrodes are closest, and the electric field is the highest (Figure 5a). The zipping electrodes displace the liquid dielectric toward the region of the shell that is not covered by electrodes. The hydrostatic pressure inside the shell increases—due to the inextensibility of the shell and the incompressibility of the liquid dielectric—and causes linear contraction of the actuator (Figure 5a,b).

The progressive zipping mechanism avoids the pull‐in instability observed in elastomeric donut HASEL actuators. Peano‐HASEL actuators require an activation voltage, whose magnitude depends on the actuator geometry, materials system, and the applied loads, but when this voltage is exceeded, the electrodes zip controllably and provide for voltage‐mediated actuation (Figure 5c). At a voltage of 10 kV, the first Peano‐HASEL actuators demonstrated linear contraction up to 10% when no load was applied. Compared to elastomeric HASEL actuators, the use of thin‐film dielectrics lowered the voltages required for actuation (Figure 5c). Depending on the application, the output force may be readily increased by stacking multiple Peano‐HASEL actuators in parallel (Figure 5d). The inherent speed of electrostatic actuation allowed for high‐speed operation, with activation times as low as 12 ms (Figure 5d). Further, the inextensible structure allowed for high power densities up to 160 W kg^−1^ without the use of resonant modes.^[^ ^15^ ^]^ Using hydrogel electrodes allowed for the creation of an actuator that was imperceptible when submerged in fluid (Figure 5e).

A central contribution of this work^[^ ^15^ ^]^ was the introduction of thin, inextensible thermoplastic films as the shell material, which facilitated the use of industrially amenable fabrication processes and materials to realize scalable, accessible, and versatile actuators. In this first work,^[^ ^15^ ^]^ the dielectric shell consisted of packaging‐grade biaxially oriented polypropylene (BOPP) films with breakdown strengths of nearly 700 kV mm^−1^, which allowed for large Maxwell stresses. Rather than relying on chemical or adhesive bonding, we could bond these films using a rapid heat‐stamping method. Further, inextensible films eliminated the need for stretchable electrodes, which often suffer from low reliability and high resistivity.^[^ ^45^ ^]^ For example, in this work, we demonstrated the use of flexible aluminum electrodes, which were industrially deposited using a physical vapor deposition process. While these electrodes were fragile and susceptible to mechanical wear and ablation (at only ≈30 nm thick), they demonstrated the potential for a broad spectrum of usable electrode materials. The total cost of materials for these actuators was ≈$0.10.^[^ ^15^ ^]^

### 3.2 Rapid Prototyping of HASEL Actuators Made from Thermoplastic Polymers

**Figure 6:**
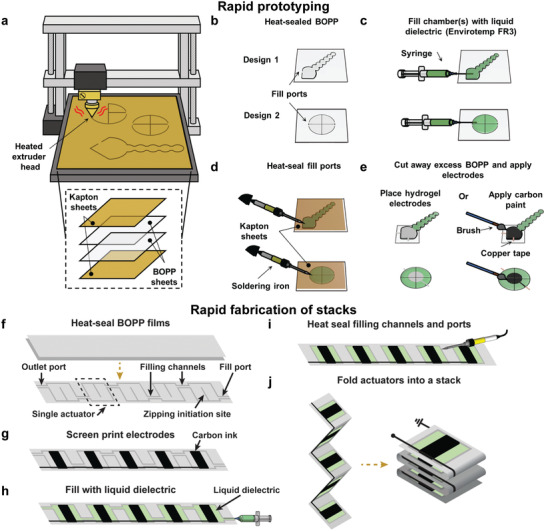
Rapid prototyping of HASEL actuators made from thermoplastic polymers. a) Two thermoplastic sheets are bonded together with a CNC heat‐sealing machine to form a shell, b) which allows the rapid fabrication of different designs. c) The shells are filled with the liquid dielectric with a syringe through a fill port. d) The fill ports of the shell are sealed with a hot soldering iron. e) Electrodes are applied to the filled actuator (alternatively electrodes may also be applied to the shell before filling). f) To rapidly fabricate a stack of actuators, two thermoplastic films are bonded together to form a strip of multiple interconnected actuators. g) Then, electrodes are screen‐printed onto the strip, and h) all actuators are filled in a single step with a liquid dielectric. i) Finally, the fill port and the connections between the individual actuators are sealed, and j) the strip is folded to form a stack of actuators. a–j) Reproduced under the terms of the CC‐BY Creative Commons Attribution 4.0 International license (https://creativecommons.org/licenses/by/4.0).^[^ ^89^ ^]^ Copyright 2019, The Authors, published by Wiley‐VCH.

The use of heat‐sealable thermoplastic films opens a large material and design freedom for the dielectric shell of HASEL actuators. To fabricate the first Peano‐HASEL actuators, we utilized a heat‐stamping method to bond thin films of BOPP into rectangular shells.^[^ ^15^ ^]^ Although this bonding method was fast, the heat stamps could not easily be adjusted to other designs, because each design required machining a new metal dye to act as the heat stamp. Quickly exploring the design freedom inherent to HASEL actuators required a tool that was well‐suited to rapidly prototype geometries of actuators. Inspired by the fabrication techniques used to created fluid‐driven soft actuators from thin films,^[^ ^85^, ^91^, ^92^ ^]^ we converted an off‐the‐shelf 3D printer into a three‐axis CNC (computer numerical control) heat‐sealing machine.^[^ ^89^ ^]^ Using commercially available computer aided design (CAD) software, the heated extruder tip of the CNC machine was programmed to trace 2D patterns to thermally bond films of thermoplastic polymers (**Figure** **6**a). This tool provided a fast and effective method of creating nearly arbitrary 2D geometries of the dielectric shell (Figure 6b). To fabricate a full actuator, the heat‐sealed shells were subsequently filled with liquid dielectrics (Figure 6c), sealed (Figure 6d), and coated with electrodes (Figure 6e). Our initial prototypes utilized familiar actuator materials such as BOPP as the dielectric shell, a vegetable‐based liquid dielectric (Envirotemp FR3), and ionically conductive hydrogels and carbon paint as electrodes (Figure 6). However, this fabrication technique is suited for a plethora of thin‐film thermoplastics (e.g., poly(ethylene terephthalate), thermoplastic polyurethane), as well as other liquid dielectrics (e.g., silicone oil, mineral oil).

The process of heat‐sealing individual shells (Figure 6a–e) is ideal for prototyping individual actuators, but it is not efficient at creating many actuators simultaneously. To address this issue, we developed a technique to rapidly fabricate multiple actuators of the same design (Figure 6f–j).^[^ ^89^ ^]^ In this technique, two thermoplastic layers are bonded to form multiple interconnected shells of actuators (Figure 6f). Screen‐printing conductive ink allows electrodes to be applied to all actuators simultaneously (Figure 6g), which mitigates the need for hand‐placed electrical connections and enables intricate electrode patterns. All the actuators are filled simultaneously though a single fill‐port (Figure 6h), thereby greatly reducing the time for filling. Then, the filling channels between the individual actuators are sealed (Figure 6i). The actuators may be folded to form a stack of mechanically and electrically connected actuators (Figure 6j). This fabrication technique may also be used to fabricate multiple, mechanically disconnected, actuators simultaneously—by cutting the actuators apart instead of folding them into a stack.

The ability to rapidly prototype and customize HASEL actuators was critical for exploring their intrinsically large design space. It allowed for optimization of existing designs of HASEL actuators and the development of actuators with entirely new types of motion. We give an overview of the current designs of thermoplastic HASEL actuators below—many of which employ the rapid prototyping method described in this section.

### 3.3 Quadrant Donut HASEL Actuator

**Figure 7:**
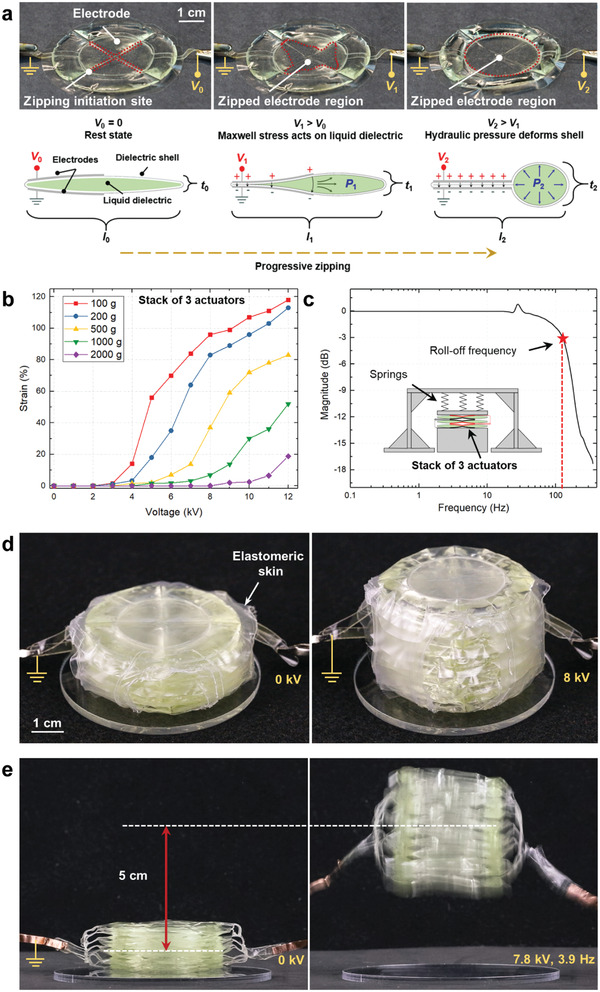
Quadrant donut HASEL actuator with progressive zipping. a) The quadrant donut HASEL actuator consists of a circular shell, which is subdivided into four equal quadrants. Circular electrodes cover the center of the actuator. When a voltage is applied to the actuator, the electrodes progressively zip from the center of the shell outward and the actuator takes a donut shape. b) The strain–voltage curves of a stack of three quadrant donut actuators do not show a pull‐in instability. c) Frequency response of a stack of three quadrant donut actuators to a sinusoidal excitation signal. d) Photographs of a stack of 11 quadrant donut HASEL actuators during actuation. e) The stack of quadrant donut HASEL actuators has a high power to weight ratio and thus can jump without requiring a power amplification mechanism. a–e) Reproduced under the terms of the CC‐BY Creative Commons Attribution 4.0 International license (https://creativecommons.org/licenses/by/4.0).^[^ ^89^ ^]^ Copyright 2019, The Authors, published by Wiley‐VCH.

With the Peano‐HASEL actuator^[^ ^15^ ^]^ made from thermoplastic shells, we had demonstrated controlled linear contraction due to progressive zipping of the electrodes (Section 3, .1). We observed that while these actuators contract in length, they also expand in thickness (Figure 5a). We used this effect to design a HASEL actuator with controllable linear expansion: the quadrant donut HASEL actuator (**Figure** **7**a).^[^ ^89^ ^]^ This actuator consists of a circular thermoplastic shell that is covered in the center on both sides with circular electrodes. A heat seal divides the shell into four discrete quadrants. This heat seal provides a zipping initiation site to prevent the pull‐in instability that occurred in the elastomeric donut HASEL actuator (Section 2, .1). Additionally, the heat seal promotes an even distribution of the liquid dielectric in the shell and consequently an equal expansion of each quadrant during actuation, which proved especially advantageous when stacking quadrant donut HASEL actuators to increase the actuation stroke.^[^ ^89^ ^]^

Stacks of three quadrant donut HASEL actuators possessed a well‐rounded performance; compared to elastomeric donut actuators (Figure 3), they exhibited larger linear expansions (up to 118% at a load of 1 N and a voltage of 12 kV) and generated more force at lower voltages (Figure 7b). Quadrant donut HASEL actuators exhibited specific energies up to 12 J kg^−1^, and a roll‐off frequency as high as 126 Hz (Figure 7c).^[^ ^89^ ^]^ We created modular units of 11 quadrant donut HASEL actuators (Figure 7d); these units were fast and powerful enough to jump upon application of a square wave voltage signal (Figure 7e). Multiple units could be stacked to further increase actuation stroke, while parallel arrays of units could be created to increase the actuation force (Section 8, .1).

### 3.4 Curling HASEL Actuators

**Figure 8:**
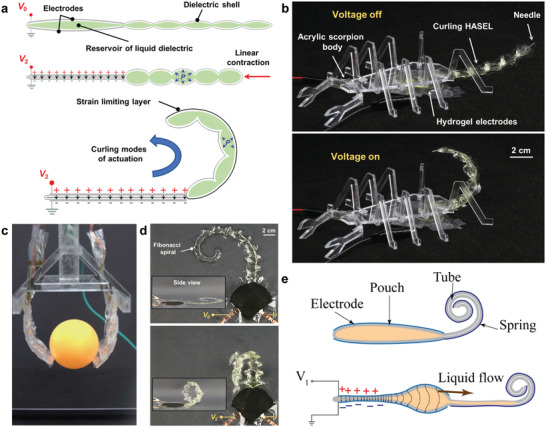
Curling HASEL actuator. a) A curling HASEL actuator consists of a reservoir of liquid dielectric connected to a corrugated shell. The reservoir is covered with electrodes. When the electrodes zip, the liquid dielectric is displaced into the corrugated shell, which contracts. Attaching a strain limiting layer to one side of the actuator leads to curling. b) Photographs of a biomimetic scorpion tail. c) Curling HASEL actuators can be used for soft grippers capable of grasping objects. d) A curling actuator shaped like a Fibonacci spiral simultaneously curls and twists. e) Using a coiled spring as a strain limiting layer creates an actuation mode that is the inverse to curling; upon activation, liquid dielectric forces the coiled spring to unravel, which causes the actuator to elongate. a,b,d) Reproduced under the terms of the CC‐BY Creative Commons Attribution 4.0 International license (https://creativecommons.org/licenses/by/4.0).^[^ ^89^ ^]^ Copyright 2019, The Authors, published by Wiley‐VCH. c) Reproduced with permission.^[^ ^94^ ^]^ Copyright 2020, Mary Ann Liebert, Inc. e) Reproduced under the terms of the CC‐BY Creative Commons Attribution 4.0 International license (https://creativecommons.org/licenses/by/4.0).^[^ ^95^ ^]^ Copyright 2019, The Authors, published by MDPI.

Motivated by hydraulic actuation found in species of arachnids,^[^ ^93^ ^]^ we prototyped bioinspired designs of HASEL actuators that contract, curl, or twist (**Figure** **8**).^[^ ^89^ ^]^ In a basic design, the shell of these actuators consists of a large, electrode‐covered chamber, which acts as a reservoir of liquid dielectric, connected to a corrugated structure of smaller chambers (Figure 8a). Upon activation, the liquid dielectric is forced from the reservoir into the corrugated structure; the radius of curvature of the corrugations decreases and the structure contracts linearly. A curling motion may be achieved by attaching a strain‐limiting layer to one side of the actuator, which prevents the corrugations from contracting on that side, and consequently leads to curling of the corrugated structure away from the strain limiting layer. These curling HASEL actuators resemble the strike of a scorpion tail (Figure 8b), demonstrating biomimetic striking velocities of 1.26 m s^−1^. More recently, Park et al.^[^ ^94^ ^]^ investigated the efficacy of curling HASEL actuators as soft robotic grippers (Figure 8c). Additional types of actuation can be realized by modifying the corrugated structure. For example, we designed an actuator in which the corrugated structure was a Fibonacci spiral (Figure 8d); when activated, this actuator simultaneously curled and twisted. Lin et al.^[^ ^95^ ^]^ developed an actuator with an inverse curling motion, wherein a coiled spring was used as the strain limiting layer. When activated, the pressurized fluid within the shell caused the spring to uncoil, mimicking the deformation of a proboscis found in butterflies (Figure 8e).

### 3.5 High‐Strain Peano‐HASEL Actuator

Peano‐HASEL actuators have shown well‐rounded muscle‐mimetic performance; however, their theoretical actuation strain is limited to 24% (15% has been achieved experimentally),^[^ ^96^ ^]^ whereas natural muscle reaches up to 40%, with typical values of 20%.^[^ ^3^ ^]^ This limit is a result of the design of Peano‐HASEL actuators (Figure 5a), wherein the electrode‐covered and the uncovered regions of the shell are aligned in the actuation direction. When the electrodes zip, only the region of the shell that is not zipped contracts (Figure 5a); in fact, the region of the shell that is zipped elongates.

**Figure 9:**
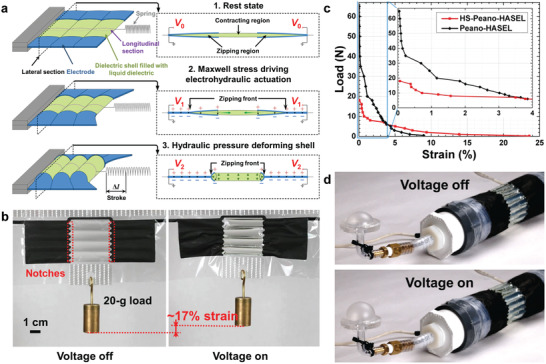
The high‐strain Peano‐HASEL actuator. a) The high‐strain Peano‐HASEL actuator consists of rectangular shells with electrodes placed on the sides of the shells. When the electrodes zip, the liquid dielectric is displaced to the center of the shells, leading to contraction of the actuator. b) Photographs of a contracting high‐strain Peano‐HASEL actuator. c) Because the electrodes are placed orthogonally to the actuation direction, the high‐strain Peano‐HASEL actuator achieves larger actuation strains than a Peano‐HASEL actuator. However, it has a comparatively smaller blocking force. d) Photographs of a cylindrical pump based on a high‐strain Peano‐HASEL actuator, which mimicked the actuation of circular muscle layers found in nature. a–d) Reproduced with permission.^[^ ^97^ ^]^ Copyright 2019, Wiley‐VCH.

In the high‐strain (HS‐)Peano‐HASEL actuator, this problem is overcome by placing the electrodes on the sides of each shell (**Figure** **9**a,b). When a voltage is applied, the electrodes zip from the side of the shell, forcing the liquid dielectric toward the center of the actuator (Figure 9a,b), which contracts. The maximum theoretical strain of the central region is 36% (when each shell forms the shape of a cylinder).^[^ ^97^ ^]^ The force–strain behavior of HS‐Peano‐HASEL actuators is similar to that of Peano‐HASEL actuators (Figure 9c), but with a higher maximum strain and a smaller blocking force. HS‐Peano‐HASEL actuators achieved up to 24% strain at a load of 0.1 N and a voltage of 10 kV (60% higher than the maximum strain recorded for Peano‐HASEL actuators).

Large actuation strains make HS‐Peano‐HASEL actuators well suited for applications that require large deformations in a compact space. One such application is a circular muscle, which is a hollow circular structure that reduces its cross‐sectional area upon activation. By wrapping a 12‐unit HS‐Peano‐HASEL actuator around a silicone tube, we fabricated a bioinspired artificial circular muscle (ACM) that acted as a pump (Figure 9d).^[^ ^97^ ^]^ Compared to a Peano‐HASEL actuator, a HS‐Peano‐HASEL actuator is favorable in this application, since it generates a larger relative change of the cross‐sectional area and thus increases the maximum flow rate of the pump. The pump generated peak pressures of 2.7 kPa and peak flow rates as high as 2.3 L min^−1^.^[^ ^97^ ^]^

### 3.6 HASEL Actuator with a Single Shell and Multiple, Individually Addressable Pairs of Electrodes

**Figure 10:**
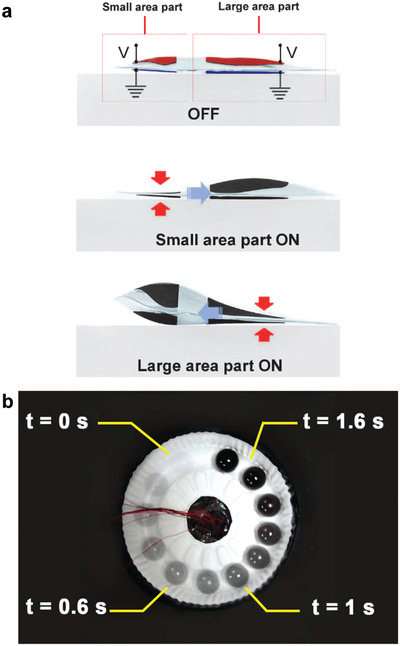
A HASEL actuator with a single shell and multiple, individually addressable pairs of electrodes. a) Schematic of an actuator that consists of a single shell that is covered with two pairs of electrodes of different size. When a voltage is applied to one electrode, the liquid dielectric is displaced causing the region of the other electrode to expand. Because the electrodes have different sizes, the force–strain characteristics depend on which pair of electrodes is activated. b) Photograph of rotary device controlled by four actuators shown in (a); the device rolls a ball in a circle. a,b) Reproduced with permission.^[^ ^98^ ^]^ Copyright 2020, IEEE.

The previous sections have elucidated the design freedom of thermoplastic HASEL actuators. Even though the discussed actuators exhibit different types of actuation and zipping modes, each individual actuator has only one controllable degree of freedom. Multiple actuators must be combined to achieve multiple controllable degrees of freedom in a single device (see Section 8, .1 for an example in which quadrant donut and curling HASEL actuators were combined into a continuum arm with an attached gripper). Recently, Kim and Cha^[^ ^98^ ^]^ have demonstrated a HASEL actuator that incorporates two individually addressable pairs of electrodes of different sizes into a single shell (**Figure** **10**a). Applying a voltage to one pair of electrodes caused this pair to zip together, which displaced the liquid dielectric and caused expansion of the shell in the region of the inactive pair of electrodes (Figure 10a). Applying the voltage to the smaller pair of electrodes caused a smaller expansion than applying the voltage to the larger pair of electrodes (i.e., the voltage–strain characteristic of each controllable degree of freedom was different). The authors exploited this asymmetric actuation for a rotary device that rolled a ball in a circle (Figure 10b).

### 3.7 Research Opportunities for HASEL Actuators Made from Thermoplastic Polymers

Moving forward, there are numerous avenues for research on thermoplastic HASEL actuators, from basic research to application‐focused metrics. A few that we believe to be promising are discussed in this section.

#### 3.7.1 Actuators with Application‐Driven Designs and New Functionalities

While initial work on thermoplastic HASEL actuators has focused on exploring fundamental actuation modes spanning linear contraction, expansion, curling, and twisting, future work will likely shift toward application‐driven designs and new functionalities. For example, integrating thermoplastic actuators with structural components may enable antagonistic groups of actuators that can support and generate both tensile and compressive forces. Further, adding elastic components into thermoplastic HASEL actuators may provide means for mechanical energy storage (and fast release), like the system of muscles and tendons in the human body. These research avenues may be supported by a systematic modeling‐based approach that improves the understanding of the electromechanical behavior of thermoplastic HASEL actuators and helps to identify design criteria (Section 5).

#### 3.7.2 New Materials and Fabrication Methods

Great potential for the improvement of the performance of thermoplastic HASEL actuators lies in the development of specialized materials. Most materials that have been used to date for thermoplastic HASEL actuators were originally developed for the packaging industry because they are inexpensive, available in large quantities, and incorporate heat‐sealing layers that aid bonding of the films. Even though these films have good mechanical properties, they were not optimized for electrical properties. Other materials such as PVDF have been optimized for high‐voltage applications (e.g., high voltage thin film capacitors) and therefore possess good electrical properties, but only possess poor mechanical properties. New materials with high dielectric constants and high dielectric breakdown strengths, as well as high mechanical strength and flexibility, would drastically expand the scope of practical applications for HASEL actuators by enabling strong and reliable actuators that may survive millions of actuation cycles.

Electrical and mechanical self‐healing performance represents another important factor in actuator lifetime; unlike elastomeric HASEL actuators, thermoplastic actuators show very limited self‐healing capabilities after dielectric breakdown, which punctures the polymer film. In designs in which electrodes are placed near the edges of the shell, dielectric breakdown often occurs through the heat seal of the shell, leading to electrical shorting of the actuator; this problem may be mitigated by using self‐clearing electrodes, which insulate the damaged region.^[^ ^53^, ^55^, ^56^ ^]^ When dielectric breakdown occurs in the liquid‐filled region of the shell, liquid dielectric begins to leak out of the pouch, a problem that may be mitigated by using an appropriate encapsulation. Development of self‐healing thin‐film layers would allow for more robust operation after dielectric breakdown events and would lead to longer lifetimes.

A multitude of polymer films with unique properties exists—with more being synthesized regularly—that cannot be heat‐sealed. The use of adhesives to form shells may expand the range of usable materials and would avoid the heat‐sealing process, which can damage the thermoplastic layers and cause premature dielectric breakdown through the heat seal and therefore decrease the maximum performance and the lifetime of actuators. Other methods of fabrication for HASEL actuators including additive manufacturing are almost unexplored and may substantially increase the design freedom.

#### 3.7.3 Studies on Lifetime and Failure Modes in Actuators

Practical implementations of HASEL actuators will benefit from studies on the lifetime and failure modes present in thermoplastic HASEL actuators. The most prevalent mode of failure for heat‐sealed systems is dielectric breakdown through the heat seal, typically near the edge of an electrode. This results in failure well below the critical electric fields measured in the films themselves. At times, this failure is preceded by repeated partial discharges through the air at the heat seal, suggesting potential electrical degradation. Methods to mitigate this failure mode could focus on eliminating partial discharges through air by implementing dielectric coatings, as observed by La et al.^[^ ^99^ ^]^ Studies aiming to understand the effects of electric field concentrations at the edges of electrodes could further enhance actuator lifetime and reliability.

#### 3.7.4 Performance under Realistic Environmental Conditions

As HASEL actuators find further application in the real world, it will be important to understand their actuation performance, lifetime, and reliability in diverse and dynamic environments. For example, the effects of humidity must be studied, as it has been shown to play a role in both the electrical and mechanical performance of dielectric elastomer actuators.^[^ ^48^, ^100^ ^]^ In addition, studies of the temperature dependence of HASEL actuators will be important for many applications such as space exploration.^[^ ^101^ ^]^

## 4 Actuation Performance of Currently Demonstrated HASEL Artificial Muscles

The performance of natural muscle, in particular mammalian skeletal muscle, is the benchmark for soft actuators—not because it excels in a specific performance metric, but because natural muscle exhibits an excellent all‐round performance (**Table** **1**). Additionally, natural muscle regenerates continuously, allowing it to actuate billions of times over the lifetime of an organism.^[^ ^4^ ^]^ Across a variety of designs, HASEL actuators have exhibited performance metrics on par with natural muscle (Table 1). To date, HASEL actuators have demonstrated actuation stresses of 0.3 MPa, contractile strains up to 24% (expanding strains up to 124%), peak strain rates of 7400% s^−1^, specific energies of 12 J kg^−1^ (70 J kg^−1^ at resonance), peak specific powers of 360 W kg^−1^ (614 W kg^−1^ at resonance), electromechanical conversion efficiencies up to 21%, and roll‐off frequencies of 126 Hz. While HASEL actuators already outperform mammalian skeletal muscle in speed and specific power, muscle still outperforms HASEL actuators slightly in actuation stress, specific energy and efficiency; only the lifetime of muscle (≈10^9^ cycles) exceeds the lifetime of HASEL actuators (≈10^6^ cycles) by a factor of ≈10^3^. Current elastomeric HASEL actuators have achieved over 10^6^ cycles without failure, while thermoplastic actuators have achieved ≈10^4^–10^5^ cycles (Table 1). More research into self‐healing materials and high‐performance dielectrics will improve the reliability of these actuators, especially in thermoplastic material systems (Sections 2, .3 and Section 3, .7). Additionally, in Section 6, we lay out a roadmap for improving the actuation stress and the energy density of HASEL actuators that may allow them to substantially exceed the values achieved by natural muscle across many performance metrics.^[^ ^96^ ^]^

**Table 1:** Comparison of current actuator performance for different types of HASEL actuators and mammalian skeletal muscle

	Linear strain [%]	Max. actuation stress [MPa]	Peak strain rate [% s^−1^]	Specific energy [J kg^−1^]	Specific power [W kg^−1^]	Efficiency [%]^a)^	Bandwidth [Hz]^b)^	Lifetime [cycles]
Mammalian skeletal muscle	20 (typ) 40 (max)^[^ ^3^ ^]^	0.35^[^ ^3^ ^]^	500^[^ ^3^ ^]^	8 (typ) 40 (max)^[^ ^4^ ^]^	50 (avg) 200 (peak)^[^ ^3^ ^]^	40^[^ ^3^ ^]^	Moderate^[^ ^16^ ^]^	10^9[^ ^4^ ^]^
Elastomeric HASEL actuators								
Donut HASEL actuator	50^[^ ^14^ ^]^	0.002^c)^	–	–	–	21^[^ ^14^ ^]^	–	>10^6[^ ^14^ ^]^
Planar HASEL actuator	124^d)^ ^[^ ^14^ ^]^	0.3^d)^ ^[^ ^14^ ^]^	–	70^d)^ ^[^ ^14^ ^]^	337^d)^ (avg) 614^d)^ (peak)^[^ ^14^ ^]^	–	–	10^5[^ ^14^ ^]^
Thermoplastic HASEL actuators								
Peano‐HASEL	15^[^ ^96^ ^]^	0.21^e)^	6100^[^ ^97^ ^]^	5^[^ ^97^ ^]^	180 (avg) 360 (peak)^[^ ^97^ ^]^	–	50^[^ ^15^ ^]^	10^4[^ ^15^ ^]^ to 10^5^ ^f)^
HS‐Peano‐HASEL	24^[^ ^97^ ^]^	0.09^g)^	2200^[^ ^97^ ^]^	4^[^ ^97^ ^]^	80 (avg) 120 (peak)^[^ ^97^ ^]^	–	40^[^ ^97^ ^]^	–
Quadrant donut HASEL	118^[^ ^89^ ^]^	0.03^h)^	7400^[^ ^89^ ^]^	12^i)^	120 (avg) 150 (peak)^[^ ^89^ ^]^	19^[^ ^89^ ^]^	126^[^ ^89^ ^]^	10^4[^ ^89^ ^]^ to 10^5^ ^f)^

^a)^ Efficiency for HASEL actuators is defined as the ratio between the mechanical output work and the expended electrical energy during one actuation cycle. Detailed descriptions of the experimental procedure to measure this full‐cycle efficiency can be found in Acome et al.^[^ ^14^ ^]^ and Mitchell et al.^[^ ^89^ ^]^

^b)^ Bandwidth for HASEL actuators is defined as the 3 dB roll‐off frequency

^c)^ Calculated from published data:^[^ ^14^ ^]^ the maximum stress was calculated by dividing the blocked force of 4 N by the circular area of the actuator where the load is applied (*r* = 2.5 cm)

^d)^ Measured at resonance

^e)^ Calculated from published data:^[^ ^96^ ^]^ the stress was calculated by dividing the measured blocking force of 45 N by the measured actuator cross‐section of 9 cm wide (pouch width) by 0.24 cm thick (thickest point in undeformed pouch)

^f)^ Unpublished data

^g)^ Calculated from published data:^[^ ^97^ ^]^ the maximum stress was calculated by dividing the measured blocking force of 18 N by the measured actuator cross‐section of 12 cm wide (pouch width) by 0.16 cm thick (thickest point in undeformed pouch)

^h)^ Calculated from published data:^[^ ^89^ ^]^ the maximum stress was calculated by dividing the maximum measured actuation force of 60 N by the circular area of the actuator where the load is applied (*r* = 2.5 cm)

^i)^ Calculated from published data:^[^ ^89^ ^]^ the specific energy was found by calculating the area under the force–displacement curve for a stack of three quadrant donut HASEL actuators operated at 12 kV, and then dividing this value by the mass of the stack (6 g).

In addition to providing excellent all‐round performance, HASEL actuators are promising candidates to replace electromagnetic motors (and other types of actuators) in bioinspired mobile robotic platforms for multiple reasons. First, compared to electromagnetic motors, HASEL actuators directly generate biomimetic linear motion, so they do not need transmission systems, which can be bulky, heavy, and lossy. Second, HASEL actuators operate using electrostatic and hydraulic principles, both of which exhibit better transduction efficiencies than other popular soft robotic technologies such as pneumatic soft actuators and thermally‐driven artificial muscles.^[^ ^4^, ^13^, ^102^ ^]^ Currently realized values of ≈20% for the full‐cycle efficiency for HASEL actuators^[^ ^14^, ^89^ ^]^ were measured with actuators that were not optimized for high efficiency (an ideal, lossless electrostatic transducer has a theoretical full‐cycle efficiency of 100%^[^ ^103^ ^]^). The full‐cycle efficiency of HASEL actuators can be improved in the future by reducing corona discharge into the surroundings and charge leakage through the dielectric layers, by using materials systems that exhibit low levels of residual charging (Section 6, .3), and by reducing mechanical losses in the shell, viscous losses in the liquid dielectric, and resistive losses in the electrodes. Additionally, it will be important to determine and improve the overall system efficiency of robotic systems driven by HASEL actuators (energy extracted from battery compared to mechanical work performed on an external load); the design of optimized high‐voltage driving electronics (Section 8, .1) and control schemes (Section 8, .3) are largely unexplored and will be fruitful areas of future research. Third, HASEL actuators have a catch state: once actuated, the electrostatic actuation principle allows for continuous force output with very little energy consumption (largely due to charge leakage through the dielectric layers). In contrast, electromagnetic motors and natural muscle consume continuous energy—electrical and chemical, respectively—to maintain an output state. Electric motors can include nonbackdrivable transmissions to provide catch states, but this approach increases complexity and weight of the system. Because of the existence of a catch state, the efficiency of robotic systems driven by HASEL actuators may even exceed the efficiency of systems driven by electromagnetic motors for sequences of motions that include holding loads at specific positions. Finally, HASEL actuators are based on inexpensive materials (e.g., the material cost for a Peano‐HASEL actuator can be ≈$0.10^[^ ^15^ ^]^), which facilitates the development of low cost robots.

## 5 Modeling of HASEL Artificial Muscles and Fundamentals of Electrohydraulic Transduction

**Figure 11:**
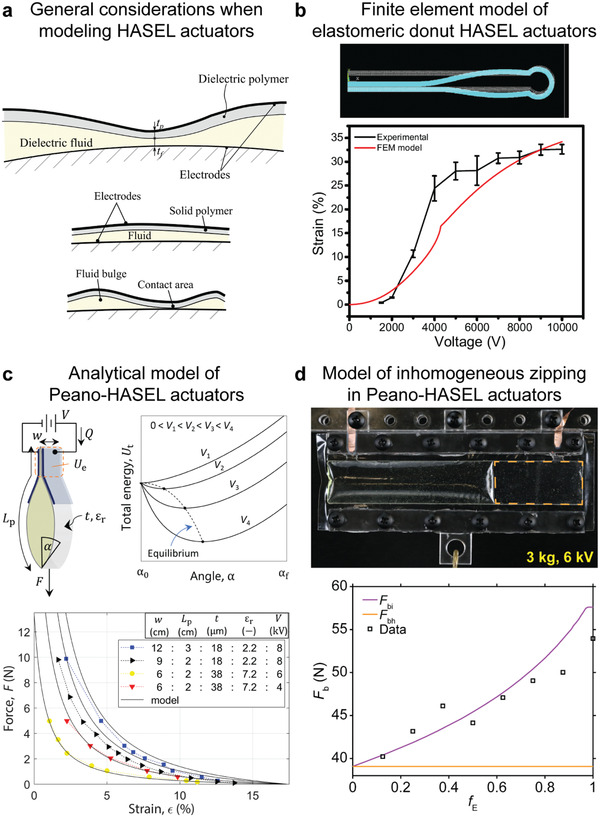
Modeling of HASEL actuators. a) The electrical structure of a HASEL actuator is a complex capacitive architecture that includes solid and liquid dielectrics of different thicknesses. A mechanical description needs to include the large deformations of the shell, contact between the walls of the shell, and fluid‐structure interaction. b) Finite element model of an elastomeric donut HASEL actuator. The model included nonlinearities due to large deformations, the hyperelastic behavior of the shell, and contacts. The electric field was calculated in the shell and the liquid dielectric. The liquid was modeled as a hydrostatic medium. c) Analytical quasi‐static model of Peano‐HASEL actuators. The force–strain curves of the actuators are calculated by minimizing the Helmholtz free energy of the system, which includes the electrical energy of the voltage supply, the electrical energy stored in the actuator, and the potential energy of the applied load. Without relying on a fitting factor, the model agrees very well with experimental results. d) At high loads near the blocking force, Peano‐HASEL actuators exhibit an instability that leads to inhomogeneous zipping (dashed rectangle indicates zipped region of the electrodes). Inhomogeneous zipping leads to an increase in the blocking force (*F*_b_) of Peano‐HASEL actuators, an effect that becomes stronger with higher electrode coverage (*f*_E_ = length of electrode/length of shell). a) Reproduced with permission.^[^ ^104^ ^]^ Copyright 2018, SPIE. b) Reproduced with permission.^[^ ^105^ ^]^ Copyright 2018, The Authors. c) Adapted with permission.^[^ ^96^ ^]^ Copyright 2019, Elsevier Ltd. d) Reproduced with permission.^[^ ^108^ ^]^ Copyright 2019, Elsevier Ltd.

It is important to develop a rigorous theoretical framework for the electromechanical behavior of HASEL actuators, because it will inform modification of materials and geometries for performance improvement in current designs and allow prediction of the behavior of entirely new designs. Moretti et al.^[^ ^104^ ^]^ described general considerations for the modeling of HASEL actuators. Though they are driven by a Maxwell stress like dielectric elastomer actuators, HASEL actuators are more difficult to describe theoretically for several reasons. The thickness of liquid dielectric between the electrodes is inhomogeneous and the electrodes are not parallel (**Figure** **11**a), which leads to inhomogeneous electric fields within the actuators. Geometric nonlinearities, such as large deflections and contact between the walls of the shells, complicate the mechanical analysis (Figure 11a). If only the quasistatic behavior is of interest, the fluid may often be modeled as an incompressible medium that transmits forces through hydrostatic pressure.^[^ ^96^, ^104^, ^105^ ^]^ However, the flow‐behavior of the liquid dielectric is important for dynamic analyses of HASEL actuators. The next sections discuss specific models that have been derived to date for HASEL actuators and outline potential future modeling efforts.

### 5.1 Models of the Elastomeric Donut HASEL Actuator

Manion et al.^[^ ^105^ ^]^ conducted a multiphysics finite element simulation of an elastomeric donut HASEL actuator in ANSYS (Figure 11b). They calculated the entire voltage–strain curve of the actuator, including the pull‐in instability, with great accuracy (Figure 11b). In the simulation, the full electric field was calculated in the actuator in order to correctly describe the electromechanical coupling, and the nonlinear material behavior of the shell was considered. However, the liquid dielectric was approximated as a hydrostatic medium without pressure gradients (i.e., the flow of the liquid dielectric during actuation was not simulated), an assumption that prevents an accurate dynamic analysis. Zamanian et al.^[^ ^106^ ^]^ performed a similar simulation in COMSOL to study the influence of geometry on the actuation performance of elastomeric donut HASEL actuators.

Because most of the deformation in an elastomeric donut HASEL occurs near the pull‐in voltage (Figures 3a and 11b), it is important to understand the physics of this instability. Panwar et al.^[^ ^80^ ^]^ and Zamanian et al.^[^ ^79^ ^]^ used multiphysics finite element models and lumped parameter models to study the onset of the pull‐in instability. They demonstrated that the onset of the pull‐in instability occurs when the electrostatic attraction between the electrodes overcomes the restoring force of the actuator and a positive feedback between the deformation of the actuator and the electrostatic forces between the electrodes sets in.^[^ ^79^, ^80^ ^]^ Panwar et al. also investigated how materials properties and electrode geometry may be modified to reduce the critical voltage for the pull‐in instability.^[^ ^80^ ^]^

### 5.2 Models of the Peano‐HASEL Actuator

When the electrodes are placed near the edge of the shell in a thermoplastic HASEL actuator, it does not exhibit a pull‐in instability; instead, when a sufficiently large voltage is applied, the electrodes zip controllably from the edge of the shell, where the electric field and the Maxwell stress are highest. The rectangular geometry of Peano‐HASEL actuators is easy to describe theoretically. The Peano‐HASEL actuator may therefore serve as a model system for multiple other types of HASEL geometries that use progressive zipping (e.g., Figures 5a and 7a). In the following paragraphs we describe the basic assumptions and results of existing models for Peano‐HASEL actuators because they give an insight into the electromechanical behavior of Peano‐HASEL actuators and related HASEL geometries.

Typically, the shell of a Peano‐HASEL actuator is much wider than long (Figure 5b), so side‐constraints can be neglected. For a wide range of loads and voltages, the electrodes of a Peano‐HASEL actuator zip approximately homogeneously from the top of the actuator toward the bottom and Peano‐HASEL actuators can be modeled as 2D (Figure 11c).^[^ ^96^, ^104^ ^]^ For the investigated shell geometries and materials, the thermoplastic films that form the shells were treated as inextensible membranes. This allows modeling the shape of the liquid‐filled region of the shell as cylinder sections and describing the shape of the actuator with a single degree of freedom: the angle α (Figure 11c).^[^ ^96^, ^104^ ^]^

We obtained analytical equations of state for Peano‐HASEL actuators by minimizing the Helmholtz free energy^[^ ^107^ ^]^ of the actuator and the voltage source with respect to α (Figure 11c).^[^ ^96^ ^]^ Because the elasticity of the shell was neglected, only the gravitational potential energy of an attached weight, the electrical energy of the voltage source, and the electrical energy stored in the electric field between the electrodes were considered. As the electric field rapidly decays in the liquid‐filled region of the shell, only the electric field in the zipped region of the electrodes was considered, which could be treated as a parallel plate capacitor.

Without a fitting factor, this simple model agreed very well with experimentally measured force–strain curves over a wide range of shell‐materials, geometries, and applied voltages (Figure 11c). These results demonstrated that the simplifying assumptions listed above are good first order approximations for the electromechanical behavior of Peano‐HASEL actuators. We therefore believe that these basic assumptions are generally good for most thermoplastic HASEL actuators. Some designs may require additional considerations, such as friction between the shell and the external load in expanding HASEL actuators.

At high loads near the blocking force, thermoplastic HASEL actuators often show an unstable zipping behavior. In the Peano‐HASEL actuator this manifests as increasingly inhomogeneous zipping until eventually a part of the electrode completely zips together whereas the remaining electrode does not zip at all (Figure 11d). To understand this behavior, we extended the model described in the previous paragraphs by splitting the actuator into two sections that can zip differently.^[^ ^108^ ^]^ The model showed that inhomogeneous zipping occurs because it is energetically favorable compared to homogeneous zipping. For Peano‐HASEL actuators, inhomogeneous zipping increases the blocking force, an effect that becomes stronger with longer electrodes (Figure 11d).^[^ ^108^ ^]^

The models for the Peano‐HASEL actuator had two notable properties: i) The electric field inside the liquid‐filled region of the shell was neglected, so the predicted force–strain curves were independent of the properties of the liquid dielectric; ii) The resulting force–strain curves were independent of the length of the shell. This property provides an avenue for drastically improving the performance of Peano‐HASEL actuators, as described in Section 6.

### 5.3 Research Opportunities for Improved Models and Fundamental Understanding of HASEL Actuators

The models for HASEL actuators described above provided a first, basic understanding of the physical principles that govern the electromechanical behavior of HASEL actuators. However, more progress is necessary to fully understand the electromechanics of HASEL actuators and to be able to use theoretical calculations as a quantitative tool for the design of new types HASEL actuators (e.g., through the use of multiphysics finite element models). Several quasistatic models exist for HASEL actuators, but their dynamic behavior has yet to be investigated. In particular, the viscosity of the liquid dielectric may strongly influence the actuation speed, but none of the models of HASEL actuators include a physically accurate representation of the flow of the liquid dielectric.

Current finite element models of elastomeric donut HASEL actuators use symmetry assumptions for the shape and the deformation of shell and most models do not include a mechanical load. Extending these finite element models may enable systematic studies into the influence of geometry and load on the pull‐in instability and the effects of hydraulic amplification. To date, there is no model for planar HASEL actuators. The Peano‐HASEL and the HS‐Peano‐HASEL actuators are the only thermoplastic HASEL actuators for which theoretical models exist,^[^ ^96^, ^97^, ^104^, ^108^ ^]^ because their rectangular geometry allows for analytical or semianalytical modeling. Actuators in which side‐constraints and the formation of wrinkles in the shell play an important role for deformation (such as the quadrant donut HASEL and the curling HASEL actuators), have not been successfully modeled as of this report. These actuators may require the use of commercial multiphysics finite element packages or the development of specialized numerical tools. Another interesting avenue of research is the development of realistic electromechanical models of small‐scale thermoplastic HASEL actuators (shell length  ≪ 1 cm), in which the bending stiffness of the shell material and the electric field in the liquid‐filled region of the shell may influence or even dominate the actuation behavior. Finally, multiphysics finite element simulations will play an important role in identifying regions of high electrical and mechanical stresses in different geometries of HASEL actuators; this knowledge can be used to optimize designs for improved performance and lifetime.

## 6 Strategies to Substantially Improve the Performance of HASEL Artificial Muscles

Here we identify several potentially high‐impact avenues for creating HASEL actuators with drastically improved performance.

### 6.1 Downscaling of Actuators

**Figure 12:**
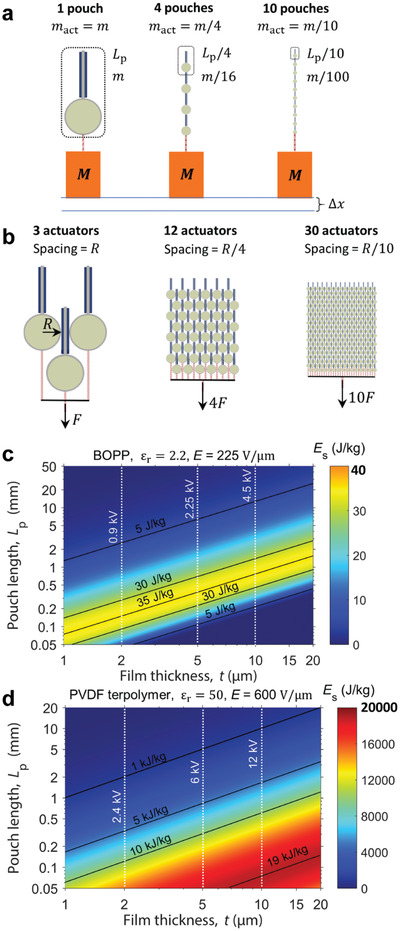
Strategies to improve the performance of HASEL actuators using the Peano‐HASEL as a model system. a) The weight of a Peano‐HASEL actuator can be decreased by replacing a single long shell with multiple short shells in series while maintaining the same force–stroke characteristics, which leads to an increase in specific energy of the actuator. b) Decreasing the shell‐length in parallel stacks of Peano‐HASEL actuators increases the actuation stress (output force divided by the cross‐sectional area of the stack) as the packing density can be increased. c) Decreasing the shell‐length increases the specific energy of Peano‐HASEL actuators until the bending stiffness of the shell reduces the actuation strain. Decreasing the film‐thickness allows the use of smaller actuation voltages but requires smaller pouch‐lengths to maintain the specific energy (indicated by black lines). d) The use of high‐performance materials with high dielectric constants and dielectric breakdown strengths may drastically improve the specific energy of HASEL actuators. a–d) Reproduced with permission.^[^ ^96^ ^]^ Copyright 2019, Elsevier Ltd.

The analytical model of the Peano‐HASEL actuator (Section 5, .2)^[^ ^96^ ^]^ showed a clear pathway for improving their performance. The model predicted that the force–strain curves of Peano‐HASEL actuators are independent of the length of the pouch (*L*_p_, **Figure** **12**a). As a consequence, an actuator that consists of a single pouch will have the same force‐stroke curves as an actuator that consists of a series short pouches with the same overall length (Figure 12a). Reducing the pouch size increases the stacking density when placing multiple actuators in parallel, as the actuator spacing (*R*, Figure 12b) scales approximately inversely with the pouch length. A higher packing density increases the force output per cross‐sectional area of the stack and consequently the actuation stress and the energy density (energy per volume). This approach works until the bending stiffness of the thermoplastic shell begins to dominate and reduce the actuation strain.^[^ ^96^ ^]^

Reducing the length of the pouch also reduces the mass (*m*, Figure 12a) of the actuator proportionally, as less liquid is required to fill the pouch, which translates to a higher specific energy (energy per mass). To estimate how much the specific energy of Peano‐HASEL actuators can be increased using this strategy we extended the analytic model by including an estimate for the bending stiffness of the thermoplastic film.^[^ ^96^ ^]^ The calculations showed that for a proven material (18‐µm‐thick BOPP film, relative permittivity ε_r_ = 2.2) at current electric fields (225 V µm^−1^) downscaling increases the specific energy to up to 35 J kg^−1^ (Figure 12c),^[^ ^96^ ^]^ which is comparable to mammalian skeletal muscle (Table 1).^[^ ^4^ ^]^ Further decreasing the pouch length leads to a decrease in specific energy, because the bending stiffness constrains the actuator. The pouch length at which the maximum specific energy occurs depends on the thickness of the film. Using smaller film‐thickness enables lower actuation voltages, but also requires a shorter pouch to obtain the same specific energy (Figure 12c).

### 6.2 Solid Dielectrics with Both High Permittivity and Dielectric Breakdown Strength

Additionally, the specific energy may be increased by using materials with higher dielectric constants and higher dielectric breakdown strengths. Materials such as PVDF terpolymers have exhibited exceptionally large Maxwell stresses when used in electrostatic devices such as an electrostatic clutch.^[^ ^109^ ^]^ A commercially available PVDF terpolymer has a relative permittivity ε_r_ = 50 and dielectric breakdown strength *E* = 600 V µm^−1^;^[^ ^72^ ^]^ using this film, the analytical model presented predicts specific energies up to 20 000 J kg^−1^ (Figure 12d) far exceeding the maximum of 40 J kg^−1^ for mammalian skeletal muscle.^[^ ^96^ ^]^

Even though the predictions of the model (Figure 12d) show the incredible potential to increase the specific energy of Peano‐HASEL actuators, the calculated numbers must be taken with caution. The model only included a simplified estimate of the bending stiffness of the shell. Additionally, side constraints and the effects of the electric field outside the zipped region of the electrode may become important at small pouch lengths. The model also does not take the mechanical strength of the actuators into account. The energy densities of HASEL actuators increase with the permittivity of the shell material, because of an increased force‐output.^[^ ^96^ ^]^ This force output may exceed the mechanical strength of the shell materials, particularly when these materials are not optimized for their mechanical properties (see also Section 3, .7.2). Finally, the feasibility and practicality of fabricating actuators with very short pouch length, and whether operating near the breakdown strength of the materials substantially reduces the lifetime of actuators will determine the possibility to create HASEL actuators with extremely high energy densities for practical applications.

### 6.3 Investigation of the Electrical Properties of Solid–Liquid Dielectric Composites

An investigation of the electrical properties of the unique solid–liquid dielectric composites introduced by HASEL actuators is of fundamental interest. In some cases, the induced electric fields in the liquid dielectric during actuation exceed the liquid's measured dielectric breakdown strength (by several times) without leading to failure in the actuator.^[^ ^96^ ^]^ There is some experimental evidence^[^ ^110^ ^]^ that shielding liquid dielectrics from direct contact with electrodes using polymer films allows those liquids to sustain electric fields above their nominal breakdown strength, but the phenomenon is not fully understood. Understanding and manipulating this effect could result in actuators that sustain higher voltages without dielectric breakdown, which would widen the operational range for HASELs to achieve higher performance and higher lifetimes. Improved understanding of the electrical properties of solid–liquid dielectric composites is particularly important in the context of using high‐permittivity solid dielectrics, because these concentrate the electric field in the liquid dielectric layer; this effect may be mitigated by using liquid dielectrics with high permittivity.

Additional studies into physical effects including interfacial charging and leakage current in solid–liquid dielectric composites could have substantial impact on actuator performance moving forward. Current thermoplastic systems experience a residual charging phenomenon. This effect leads to undesirable characteristics, such as changes in actuator strain over time when a constant voltage is applied to the electrodes, and residual actuation after the voltage is turned off. To mitigate this effect, most thermoplastic HASEL actuators require the signal polarity to be reversed every cycle,^[^ ^15^ ^]^ which increases control complexity and electronics cost. The generation, accumulation, and diffusion of space charges in polymers that are subject to high electric fields are well‐known phenomena.^[^ ^111^, ^112^ ^]^ Understanding those phenomena in solid–liquid composites may lead to the identification of new materials systems that could reduce or eliminate this effect in thermoplastic HASEL actuators, which will likely increase both the reliability and electromechanical efficiency of these systems.

## 7 Soft, Electrostatic Transducers Related to HASEL Artificial Muscles

In this section we describe several technologies that are closely related to HASEL artificial muscles. In order to provide a holistic view of the core aspects of HASELs and to clearly describe similarities to and differences from other technologies, we first provide a comprehensive definition of what we consider to be a HASEL. We consider the current scope of the HASEL technology to encompass electrohydraulic transducers with the following properties: i) They are closed systems in which a deformable structure—which can be a combination of stretchable, flexible, or rigid materials—encloses a dielectric fluid (liquid or gas); ii) Electrostatic fields are applied across the dielectric fluid allowing it to act as an electrically self‐healing layer that improves the reliability of HASEL actuators; iii) The dielectric fluid plays an important role in the transmission of forces and its redistribution drives shape change in the structure; iv) HASEL transducers do not rely on a passive substrate to function and may serve as artificial muscles in robotic systems. Below, we discuss several closely related technologies that exhibit some of these characteristics (DEAs and hydrostatically coupled DEAs were already discussed in Section 1.1); each technology provides unique benefits and drawbacks compared to HASEL transducers. We expect that research in these areas will benefit from overlap and exchange of ideas to further these technologies alongside HASEL artificial muscles.

### 7.1 Zipping Dielectric Elastomer Actuators

**Figure 13:**
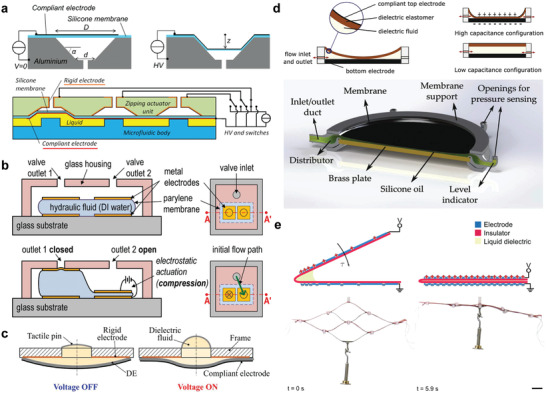
Electrostatic transducers related to HASEL actuators. a) Zipping dielectric elastomer actuators use the electrostatic zipping of a stretchable membrane onto a rigid substrate to generate large out‐of‐plane deformation. Zipping dielectric elastomer actuators have been used in peristaltic pumps. b) Electrostatic microhydraulic actuators are integrated with a rigid substrate and use electrostatic attraction to deform liquid‐filled parylene shells. Like HASEL actuators, these actuators use the principles of hydraulic amplification. Electrostatic microhydraulic actuators have been used as microvalves and micropistons. c) Electrohydraulic actuation principles have been used to create a tactile display. As a voltage is applied, electrostatic zipping of an elastomeric membrane onto a rigid substrate causes expansion of a deformable tactile dot. d) Electrohydraulic transduction principles have been used to create generators that harvest electrical energy from a periodic flow of liquid. For these devices, a stretchable membrane covers a circular chamber that is filled with a liquid dielectric. When a voltage is applied, the membrane zips onto the floor of the chamber. Pumping a liquid dielectric into and out of the chamber, these devices operate as electrostatic generators. e) In electroribbon actuators, electrostatic zipping deforms flexible ribbons to generate motion. A thin film of liquid dielectric covers the ribbons in the zipping region to improve force output. Since electroribbons are open structures, where the space between ribbons is mainly filled with air, they are lightweight and have the potential for high specific energies. a) Reproduced with permission.^[^ ^41^ ^]^ Copyright 2013, IOP Publishing Ltd. b) Reproduced with permission.^[^ ^115^ ^]^ Copyright 2016, IEEE. c) Reproduced with permission.^[^ ^117^ ^]^ Copyright 2019, SPIE. d) Reproduced under the terms of the CC‐BY Creative Commons Attibution 3.0 Unported license https://creativecommons.org/by/3.0).^[^ ^119^ ^]^ Copyright 2017, IOP Publishing Ltd. e) Reproduced with permission.^[^ ^120^ ^]^ Copyright 2018, The Authors, published by AAAS.

The concept of electrostatic zipping has been applied to dielectric elastomer actuators.^[^ ^40^, ^41^, ^113^ ^]^ These devices—known as zipping dielectric elastomer actuators (zipping DEAs)—use an elastomeric membrane with a stretchable electrode on one side, and a rigid substrate along with a fixed electrode on the other side (**Figure** **13**a). Application of voltage causes progressive electrostatic zipping of the stretchable membrane onto the rigid substrate, leading to large out‐of‐plane deformation of the membrane. An advantage of these actuators compared to regular DEAs is that they can be designed such that the electric field is not applied across the elastomeric membrane, and as such their performance can be independent of the electrical properties of the elastomeric membrane.^[^ ^113^ ^]^ Zipping DEAs have been used in peristaltic pumps for liquids (Figure 13a). Depending on the implementation, zipping DEAs can either apply an electric field across the fluid being pumped^[^ ^40^ ^]^ or avoid electric fields in the pumping medium;^[^ ^41^ ^]^ in the latter case, conductive fluids (e.g., biological fluids) can be transported. As with HASEL actuators, zipping DEAs are able to leverage high‐performance thin‐film dielectrics in order to increase actuation forces or decrease operating voltages.^[^ ^41^ ^]^

### 7.2 Electrohydraulic Actuators Relying on Passive Substrates

Electrostatic zipping mechanisms have been used in microelectromechanical systems (MEMS) for decades, as the small scale allows for reduced operating voltage and increased dielectric breakdown fields.^[^ ^114^ ^]^ One MEMS technology that leverages many of the same principles as HASEL actuators is the electrostatic microhydraulic actuator.^[^ ^115^ ^]^ These MEMS devices are made from a parylene shell filled with an insulating liquid, connected to a rigid substrate (Figure 13b). Electrodes are placed on either side of the shell, and electrostatic forces are used to deform the shell using the principles of hydraulic amplification. Sadeghi et al.^[^ ^115^ ^]^ demonstrated microvalves and micropistons using these devices. Interestingly, due to their small scale, these actuators are able to use high permittivity liquids such as deionized water as a dielectric to increase the output forces—an established practice in MEMS.^[^ ^116^ ^]^ While the operating principles of these devices is functionally similar to HASEL actuators, their reliance on a passive substrate makes them unsuitable as artificial muscles for robotic systems.

More recently, these principles have been demonstrated on a larger scale with a focus on haptic displays (e.g., active Braille), as shown in Figure 13c.^[^ ^117^ ^]^ As a voltage is applied to the device, electrostatic zipping of a dielectric membrane onto a passive substrate causes expansion of a deformable tactile “dot.” In an analogy to hydraulic pistons, these devices are able to leverage principles of hydraulic amplification based on the electrode area, amount of liquid fill, and the size of the tactile dot to influence the actuation characteristics. Han et al. demonstrated a similar electrohydraulic device for a haptic display,^[^ ^118^ ^]^ which generated perceptible forces from DC to 200 Hz.

### 7.3 Electrostatic Generators Based on Electrohydraulic Transduction

Duranti et al.^[^ ^119^ ^]^ introduced an electrostatic generator, where an electrode‐clad stretchable membrane covers a circular chamber that houses a rigid bottom electrode. This chamber is then filled with a liquid dielectric (Figure 13d). When a voltage is applied between the electrodes, the membrane zips toward the bottom electrode onto the floor of the chamber. Pumping a liquid dielectric into and out of the chamber provides mechanical‐to‐electrical energy transduction: when the membrane is fully zipped onto the bottom electrode using electrostatic forces, liquid is pumped into the chamber to mechanically separate the electrodes and to increase the electrical energy of the system.^[^ ^119^ ^]^ Unlike HASEL actuators, fluid is pumped into and out of the chamber using an external system (a syringe driven by a linear motor in this case). As with HASEL actuators, these devices could leverage a wide materials space, with electrodes and solid dielectrics ranging from stretchable to rigid.

### 7.4 Electro‐Ribbon and Electro‐Origami actuators

Electro‐ribbon and electro‐origami actuators were introduced by Taghavi et al. in late 2018.^[^ ^120^ ^]^ In these devices, flexible dielectric layers and electrode pairs create an open zipping structure (Figure 13e). A bead of liquid dielectric between the electrodes increases the electrostatic attraction—this liquid is held in place during actuation by a combination of surface tension and dielectrophoretic forces. When a voltage is applied the dielectric layers zip together to cause actuation. Electro‐origami actuators demonstrated substantial versatility in design implementation, with a variety of materials and configurations possible, including stacked structures (Figure 13e, bottom). With mostly air between the electrodes, these actuators are lightweight and have the potential to readily reach high specific energies when used as artificial muscles; however, due to the open structure, they do not benefit from the principles of hydraulic amplification, in contrast to HASEL actuators. Additionally, the open structure appeared to reduce the actuation speed in the case of multifold origami structures, and it requires periodic replacement of the liquid dielectric.

## 8 Driving Electronics and Control of HASEL Artificial Muscles

To this point, this progress report has not discussed the implementation of HASEL actuators in robotic systems. In practice, robotic systems with multiple degrees of freedom require independently addressable actuators, and appropriate sensors and control systems. This section describes driving electronics and controls for HASEL‐driven robotic systems including a discussion of high voltage safety.

### 8.1 High‐Voltage Electronics for Untethered Robotic Systems

**Figure 14:**
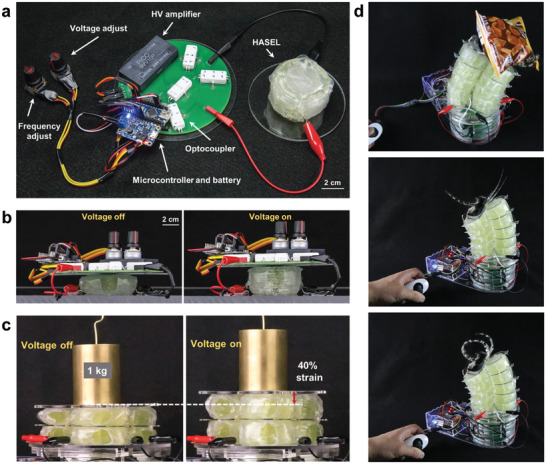
High‐voltage driving electronics for untethered soft robots based on HASEL actuators. a) A proof‐of‐concept high‐voltage power supply for untethered operation of HASEL actuators. This circuit used only off‐the‐shelf components; input power was provided by a lithium‐ion battery and the circuit was controlled with an Arduino microcontroller. The high voltages required for actuation were generated by a miniature high‐voltage amplifier, and optocouplers were used as high‐speed, high‐voltage switches to charge and discharge the actuator. b) The weight of the power supply including the battery was ≈100 g, so it was easily lifted by a stack of quadrant donut HASEL actuators operated at 8 kV. c) The power supply was used to actuate an array of three stacks of 22 actuators, which achieved 40% strain under a 1 kg load under an applied voltage of 8 kV. d) Photographs of an untethered, multiple‐degree‐of‐freedom manipulator controlled by a joystick. The soft continuum actuator consisted of three stacks of 55 quadrant donut HASEL actuators, and the design of the gripper was based on curling HASEL actuators. a–d) Reproduced under the terms of the CC‐BY Creative Commons Attribution 4.0 International license (https://creativecommons/licenses/by/4.0).^[^ ^89^ ^]^ Copyright 2019, The Authors, published by Wiley‐VCH.

HASEL actuators are particularly promising for untethered robotic systems as they are electrically driven, power‐dense, and efficient.^[^ ^14^, ^15^, ^89^ ^]^ The field of DEAs, which offer similar advantages for untethered soft robots as those listed above, has developed numerous portable high voltage power supplies (HVPS) for untethered electrostatic actuators.^[^ ^121^, ^122^, ^123^ ^]^ Taking inspiration from these works and utilizing only off‐the‐shelf components, we created a battery powered HVPS for untethered operation of HASEL actuators (**Figure** **14**a).^[^ ^89^ ^]^ This proof‐of‐concept HVPS weighed only ≈100 g and could fit into the palm of a hand. A stack of quadrant donut HASEL actuators driven by the HVPS could readily lift the power supply (including the battery), completely untethered from any external energy source (Figure 14b).^[^ ^89^ ^]^ Further, the HVPS could drive an array of three stacks of 22 quadrant donut HASEL actuators that achieved 40% strain under a 1 kg load (Figure 14c). For more advanced robotic applications which require multiple degrees of freedom, these HVPSs can be connected in parallel to create a multi‐channel driving system. We used this strategy to create a four‐channel HVPS capable of driving an untethered soft robotic manipulator (Figure 14d).^[^ ^89^ ^]^ The manipulator could grasp and reposition delicate objects, highlighting the wide potential for practical implementations of HASEL actuators in versatile, untethered robots.

### 8.2 High‐Voltage Safety

High‐voltage safety must be considered for HASEL actuators and their driving electronics. Important metrics to consider are the capacitance of the actuators, the driving voltage, the charging and discharging currents, the duration of exposure to high voltage, as well as the equivalent series resistance of the system (actuators, power supply, and the human body).^[^ ^124^ ^]^ As an example, we consider the case where an actuator is charged, disconnected from the power supply, and then accidentally discharged through contact with a human hand. At a driving voltage of 10 kV and a very conservative estimate of 500 Ω (a calloused, dry human hand can have a resistance of >100 000 Ω)^[^ ^125^ ^]^ for the equivalent series resistance of the electrodes and the human skin, accidental contact with a HASEL actuator is safe if its capacitance is below a critical value of 80 nF (according to the Underwriters Laboratories (UL) safety standard).^[^ ^124^ ^]^ Considering the above safety standard, the entire soft robotic manipulator shown in Figure 14d was safe, because it had a maximum capacitance of only ≈30 nF.^[^ ^89^ ^]^ When the actuator is connected to an activated power supply, the maximum output current of the HVPS also has to be considered for the safety of the system; for the soft robotic manipulator (Figure 14d), we limited the maximum current supplied by the HVPS to 0.3 mA, which is in a safe range.^[^ ^124^ ^]^ It is important to note that limiting the current can restrict the dynamic response of the actuator; therefore, the speed of actuation and safety requirements need to be carefully weighed when designing systems of HASEL actuators. Moreover, to safely operate large robotic systems with high‐speed HASEL actuators, one can design such systems with electrically independent subsystems, where each subsystem achieves the desired actuation speed while adhering to the safety considerations discussed above.

### 8.3 Deformation Sensing and Control for HASEL Actuators

**Figure 15:**
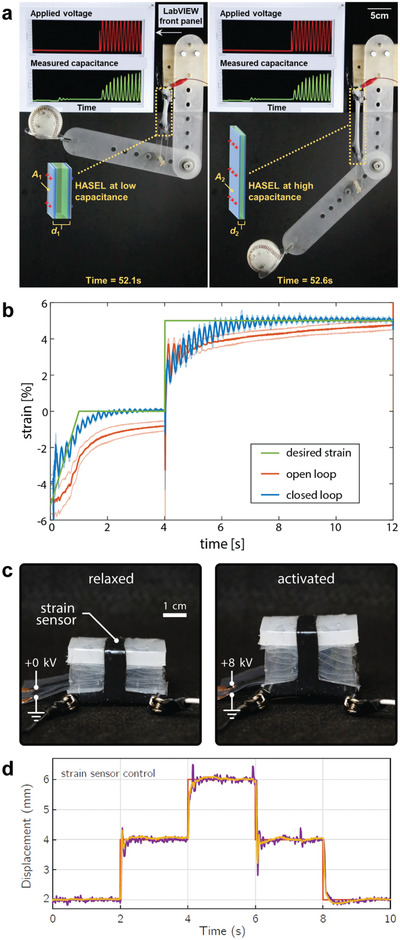
Deformation sensing and control for HASEL actuators. a) As HASEL actuators are deformable capacitors, monitoring their capacitance can be used to self‐sense deformation (see Figure 2). b) Example of closed‐loop control of a planar HASEL actuator for displacement control using self‐sensing to measure deformation. c) An external, stretchable, capacitive sensor is used to measure the deformation of a stack of expanding HASEL actuators. d) Example of closed‐loop control for displacement steps, of the actuators shown in (c) using the external capacitive sensor to measure deformation. a) Adapted with permission.^[^ ^14^ ^]^ Copyright 2018, AAAS. b) Reproduced with permission.^[^ ^126^ ^]^ Copyright 2018, IEEE. c) Reproduced with permission.^[^ ^127^ ^]^ Copyright 2020, IEEE. d) Reproduced with permission.^[^ ^127^ ^]^ Copyright 2020, IEEE.

Sensing and control of soft robots and artificial muscles is fundamentally different from approaches used in traditional rigid robotic systems.^[^ ^22^ ^]^ HASEL actuators can be closely integrated with control circuits as they are powered electrically and feature self‐sensing. Self‐sensing makes it possible to directly monitor deformation of the actuator without the use of additional sensors (Figure 2d). To demonstrate these self‐sensing capabilities, we superimposed an AC voltage signal onto the high voltage driving signal of a planar HASEL actuator to both measure its capacitance and drive the actuator via the same two electrical connections (**Figure** **15**a).^[^ ^14^ ^]^ The required AC voltage signal was about one order of magnitude smaller than the voltage required for actuation and therefore did not influence the motion.

In general, the force–displacement curves for both elastomeric and thermoplastic HASEL actuators are nonlinear, which complicates the development of an effective controller. However, for small variations in strain around an equilibrium, HASEL actuators can be treated as linear. Under this assumption, Schunk et al.^[^ ^126^ ^]^ developed a proportional‐integral (PI) controller for a planar HASEL actuator that used self‐sensing to measure the deformation. For the conditions tested, the planar HASEL actuator behaved like a linear 2nd order system, which permitted the use of a linear controller. For a step input, closed‐loop operation with the PI controller resulted in a reduced rise time and lower steady‐state error compared to open loop control, although small oscillations were present in the steady‐state response (Figure 15b).^[^ ^126^ ^]^

Another approach to measure the deformation of HASEL actuators is the use of external sensors. Johnson et al.^[^ ^127^ ^]^ developed an elastomeric, capacitive sensor skin that was wrapped around a stack of rectangular thermoplastic HASEL actuators (Figure 15c). Upon activation, the stack expanded, and the sensor‐skin stretched resulting in a change of capacitance. The capacitance of the sensor‐skin was measured with a low‐voltage circuit, that was separate from the high‐voltage electronics. Johnson et al.^[^ ^127^ ^]^ developed a proportional‐integral‐derivative (PID) controller that relied on feedback from the soft sensor‐skin. The performance of this controller was demonstrated for step inputs of the displacement (Figure 15d). Depending on the operation conditions, the controller reduced the transition times between the different states by 60–80% compared to open loop control.

### 8.4 Research Opportunities for Driving Electronics and Control of HASEL Artificial Muscles

The realization of highly versatile and adaptable HASEL‐driven robots with many degrees of freedom will require great strides in the development of driving electronics and controls. Off‐the‐shelf components that produce voltages on the order of 10 kV can be bulky and cost prohibitive. These drawbacks could be overcome by designing integrated high voltage circuits that are specific to the electrical requirements of HASEL actuators. Reducing the operating voltage of HASEL actuators to <500 V (for example, by using high‐performance dielectrics, Section 6) could lead to a dramatic leap in the functionality and compactness of the driving electronics, since well‐developed circuitry for piezoelectric actuators could be exploited.^[^ ^128^, ^129^ ^]^ However, large arrays of HASEL actuators with high energy density will require considerably more current than a few milliamperes when operated at their peak speeds; and so, electrical safety will need to be reconsidered for these systems. Furthermore, techniques like matrix addressing^[^ ^130^ ^]^ could be utilized regardless of the required voltage to create a compact electronics package capable of independently addressing hundreds or thousands of actuators, though such systems would require a complex network of high voltage electrical connections that would need to withstand high potential differences between adjacent cables.

Work to date on self‐sensing and closed‐loop control of HASEL actuators has been promising. However, reliable, low‐cost, and miniaturized self‐sensing circuits have not yet been demonstrated for HASEL actuators; this future research can draw inspiration from previous work in the area of DEAs.^[^ ^38^, ^131^, ^132^ ^]^ Moreover, soft logic developed for DEAs^[^ ^133^, ^134^, ^135^ ^]^ may be extended to HASEL‐based robots to couple actuation, sensing, and local feedback control in an all‐soft‐matter architecture, closely replicating the cohesive functionality of the muscular and nervous systems.

Gifting soft robots with extended periods of untethered mobility necessitates extremely efficient electronics for actuation, sensing, and computation.^[^ ^32^ ^]^ HASEL actuators have demonstrated high full‐cycle efficiency and because they are electrically driven actuators they are particularly well‐suited for creating robots with high system efficiency. Charge recovery circuitry inspired by the field of piezoelectric actuators^[^ ^136^ ^]^ could be used to improve the system efficiency by recovering the electrical energy discharged from HASEL actuators when they are deactivated. More advanced concepts may utilize the ability of HASEL actuators to operate as electrostatic generators to convert mechanical energy from environmental perturbations into usable electricity.

## 9 Conclusions and Outlook

Only two years have passed since the first two publications on HASEL artificial muscles;^[^ ^14^, ^15^ ^]^ in this short time span, the understanding of HASEL actuators has increased dramatically—from fabrication, materials, design, and modeling to understanding of the fundamentals of electrohydraulic transduction. Electrohydraulic HASEL actuators are extremely versatile as they achieve all three basic modes of actuation (expansion, contraction, and rotation), they feature the ability to self‐sense their deformation state, and they can be made from a variety of material systems in many different form‐factors and sizes. Current designs of HASEL actuators exhibit well‐rounded, muscle‐like performance which already makes them a viable alternative to other types of artificial muscle. Moreover, HASEL artificial muscles are a young field of research and a technology with a clearly evident potential for drastically improved performance. Realizing the full potential of HASEL artificial muscles will require pushing the limits of current knowledge and technology, which will require creative contributions from researchers including materials scientists, physicists, chemists, mechanical and electrical engineers, and roboticists for many years to come. This progress report described some immediate research opportunities that we can foresee, but we expect that with time many other interesting research questions will arise.

Considering their wide potential for further improvement, HASEL actuators are a prime candidate to become the first technology where all performance metrics match or exceed the capabilities of mammalian skeletal muscle, thus solving the centuries‐old grand challenge dating back to Robert Hooke, to replicate the remarkable properties of natural muscle. Such artificial muscles will be key building blocks of future robotic systems that mimic the rich multifunctionality of organisms found in nature. These bioinspired robotic systems will increasingly be integrated into our lives, spanning all the way from robots for disaster response to medical and consumer robotics, as well as to wearable robotic devices and lifelike prostheses. Even though there is still some way to go, we are approaching a world in which human and machine are seamlessly integrated to surpass the physical and cognitive limitations of the human body, as seen today only in science‐fiction movies. It is not a question of if this will happen, but rather when.

## Conflict of Interest

C.K., S.K.M., N.K., and E.A. are listed as inventors on a U.S. provisional patent application (62/813266) and PCT applications (PCT/US2018/023797 and PCT/US19/020568) which cover fundamentals and basic designs of HASEL actuators as well as methods of fabrication. C.K. S.K.M, and E.A. are listed as inventors on a U.S. provisional patent application (62/886820) that details use of HASEL actuators as pumps. C.K. and S.K.M. are listed as inventors on a U.S. provisional patent application (62/946317) that describes high strain Peano‐HASEL actuators. C.K., S.K.M., N.K., and P.R. are listed as inventors on a PCT application (PCT/US20/20978) that details composite dielectric structures for HASEL actuators. C.K., S.K.M., N.K., and E.A. are cofounders of Artimus Robotics, a start‐up company commercializing HASEL actuators.
